# A MYST Histone Acetyltransferase Modulates Conidia Development and Secondary Metabolism in *Pestalotiopsis microspora*, a Taxol Producer

**DOI:** 10.1038/s41598-018-25983-8

**Published:** 2018-05-29

**Authors:** Qian Zhang, Oren Akhberdi, Dongsheng Wei, Longfei Chen, Heng Liu, Dan Wang, Xiaoran Hao, Xudong Zhu

**Affiliations:** 1 State Key Program of Microbiology and Department of Microbiology, College of Life Sciences, Nankai University, Tianjin, 300071 P.R. China; 2 Beijing Key Laboratory of Genetic Engineering Drug and Biotechnology, Institution of Biochemistry and Molecular Biology, College of Life Sciences, Beijing Normal University, Beijing, 100875 P.R. China

## Abstract

Reverse genetics is a promising strategy for elucidating the regulatory mechanisms involved in secondary metabolism and development in fungi. Previous studies have demonstrated the key role of histone acetyltransferases in transcriptional regulation. Here, we identified a MYST family histone acetyltransferase encoding gene, *mst2*, in the filamentous fungus *Pestalotiopsis microspora* NK17 and revealed its role in development and secondary metabolism. The gene *mst2* showed temporal expression that corresponded to the conidiation process in the wild-type strain. Deletion of *mst2* resulted in serious growth retardation and impaired conidial development, *e.g*., a delay and reduced capacity of conidiation and aberrant conidia. Overexpression of *mst2* triggered earlier conidiation and higher conidial production. Additionally, deletion of *mst2* led to abnormal germination of the conidia and caused cell wall defects. Most significantly, by HPLC profiling, we found that loss of *mst2* diminished the production of secondary metabolites in the fungus. Our data suggest that *mst2* may function as a general mediator in growth, secondary metabolism and morphological development.

## Introduction

Filamentous fungi are biochemically prolific organisms in terms of the production of structurally diverse, low-molecular-mass compounds known as secondary metabolites (SMs). Many fungi-produced SMs possess antibiotic, anticancer and anti-viral bioactivities^1,2^, and therefore, these compounds are attractive to pharmaceutical researchers. In recent decades, fungal genome sequence information has further reinforced the view that the production capacity of SMs in fungi is grossly underestimated. This underestimation is because a fair portion of the fungal genome that is predictably involved in the biosynthesis of SMs is silent under routine laboratory conditions^3–5^. Hence, understanding the underlying mechanism of gene expression regulation is necessary to find novel natural products and improve the yield of known ones. As a matter of fact, diverse approaches to stimulating gene expression in fungi have been successfully applied, such as by the overexpression of transcription factors, swapping of promotor sequences, or co-cultivation with bacteria^4,6–8^.

The chromatin structure of eukaryotic chromosomes plays a fundamental role in the control of gene expression^9,10^. For instance, the acetylation of histone proteins on H3 has a broad effect on the biosynthesis of secondary metabolites and development in *Aspergillus nidulans* and other fungi^11,12^. A reduction in heterochromatin marks leads to higher secondary metabolite production in *Fusarium* species^13^ and the synthesis of novel metabolites in *A. nidulans*^6^. The overexpression of *A. nidulans* histone acetyltransferase (HAT) Esa A resulted in increased SMs production^14^. The *Saccharomyces cerevisiae* SAGA complex, in which Gcn5 is one of the subunits with HAT activity, is involved in the transcriptional regulation of 12% of the yeast genome^15^, and a third of that 12% is downregulated and two-thirds are upregulated in ΔGCN5 mutants^16^, suggesting a double-faceted role of HATs in transcription regulation. GcnE, a Gcn5 equivalent histone acetyltransferase in *A. nidulans*, regulates asexual development^17^. Despite the tremendous progress that has been achieved, our knowledge of gene regulation by histone modification is still limited and incomplete in filamentous fungi, given that fungi are such a diverse group of organisms and that the enzymes involved in histone modification are complex.

**Figure 1:**
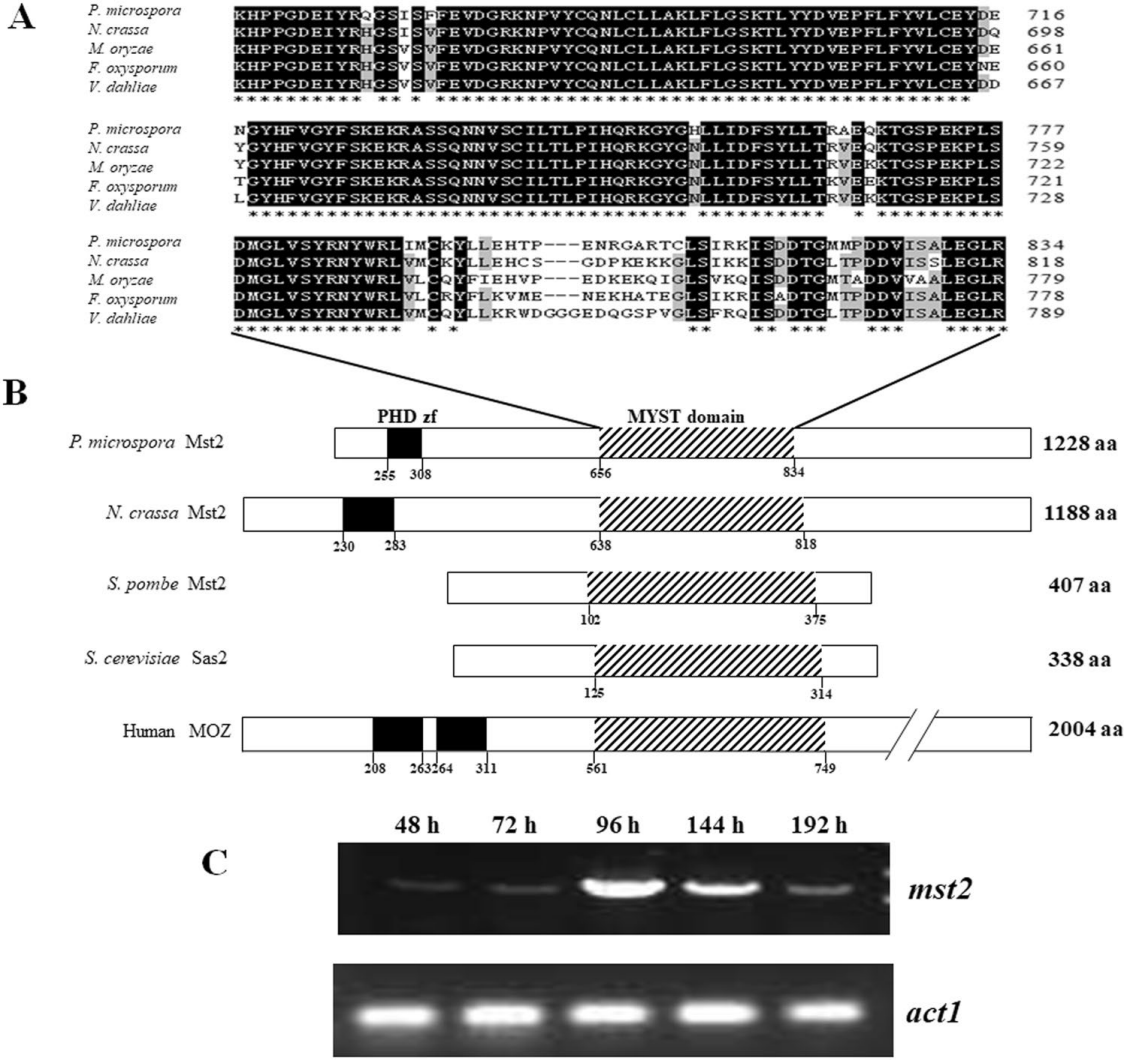
Characterization of *P. microspora* NK17 Mst2p. (**A**) Amino acid sequence alignment of the MYST domain of Mst2ps from the fungi *P. microspora* NK17 (GenBank No. KX268363), *Neurospora crassa* OR74A (EAA30995.3), *Magnaporthe oryzae* (ELQ37092.1), *Fusarium oxysporum* (ENH61771.1) and *Verticillium dahlia* (XP_009654663.1). The consensus amino acids are indicated by an asterisk (*) below the sequences, whereas the conserved amino acids are shown in grey. (**B**) A schematic comparison of Mst2p homologs from different species of fungi. The size of each HAT is indicated on the right, and the locations of the motifs are numbered. The black boxes represent PHD (plant homeodomains) zinc fingers. The MYST domain is indicated by the lined box. (**C**) Reverse transcription PCR analysis for *mst2* expression. The highest level of transcriptional expression of *mst2* was detected at 96 h. The mRNA of the actin-coding gene *act1* was used as an internal control.

The endophytic fungus *P. microspora* NK17 was recently isolated by our laboratory as a producer of a Taxol-like molecule^18^. In addition to another interesting small polyketide, pestalotiollide B, which is structurally analogous to the inhibitor of cholesterol ester transfer protein (CETP), dibenzodioxocinones were recently isolated from cultures of this strain^19^. As one of our goals is to understand the biosynthesis of SMs in this fungus, we have created a mutant library with the strategy of genome-wide mutagenesis by *Agrobacterium tumefaciens*-mediated insertion of the T-DNA in NK17^19,20^. One mutant producing less pigment with a lighter colony on agar was characterized to determine the molecular basis of this phenotype. We identified the disrupted gene, which encoded a HAT of the MYST family (Moz-Ybf2/Sas3-Sas2-Tip60) and was designated *mst2* in the study (there are three homologues in the genome). Its protein product has an MYST domain that shares high homology with SpMst2p (*Schizosaccharomyces pombe*) and other fungal Mst2ps (Fig. 1). Of the MYST family HATs in *S. pombe*, SpMst2p influences telomere structure for regional gene silencing^21^. ScSas2 and ScSas3, homologues of SpMst2p, have a similar function and are found in the baker’s yeast *S. cerevisiae*^22^. The human counterparts of MYST members include MOZ protein (monocytic leukaemia zinc finger protein), which is involved in oncogenic transformations leading to leukaemia, and Tip60, which is associated with the action of HIV^23^. In contrast, articles on the function of MYST family HATs in filamentous fungi are scarce, although fungal genome sequencing projects suggest that they are widely present in the kingdom. We describe here some important functions of MYST family member *mst2* in *P. microspora* NK17, including its critical roles in the development of conidia, fungicide resistance and the biosynthesis of secondary metabolites.

## Results

### *P. microspora* NK17 has three distinct MYST HATs

The genome of *P. microspora* NK17 has been sequenced by BGI (Shenzhen, China; http://www.genomics.cn/index). A series of BLAST searches using MYST domain sequences revealed that NK17 has three genes, GME6965_g, GME8786_g and GME6006_g, with high similarity to HATs in the MYST family, which were designated *mst1*, *mst2* and *mst3*, respectively. At the amino acid sequence level, Mst1p shares the highest similarity to Esa1p, which was essential for survival in baker’s yeast and in *S. pombe*^21,24^. Trying to delete *mst1* in *P. microspora* NK17 failed, suggesting it is an essential gene for NK17 survival.

The ORF *mst2* (GenBank no. KX268363) consists of 3,808 bp with three exons and two introns and encodes a predicted protein of 1,228 amino acids, which is significantly longer than other Mst2 protein sequences^21,25^. Structural analysis suggested that this protein contains an MYST/MOZ/SAS domain (656–834 amino acids), which is a characteristic domain of HATs in the MYST family, and a PHD zinc finger domain (255–308 amino acids), which is found in a variety of eukaryotic transcriptional factors involved in chromatin dynamics (Fig. 1). In addition, reverse transcription-PCR (RT-PCR) amplification of the mRNA of *mst2* suggested that the expression of *mst2* was culture time-dependent (Fig. 1C). During the first 72 h of vegetative growth, only a basal level of mRNA was detected, whereas robust expression of *mst2* was observed at 96 h when conidiation of the fungus supposedly started based on our experience^26^, implicating a role of Mst2p in developmental processes in NK17.

The third MYST HAT, Mst3p, shares highest similarity to *S. cerevisiae* Sas2p and Sas3p but is divergent substantially from Mst2p in terms of its peptide sequence. Characterization of Mst3p is underway. When the gene was deleted, few phenotypic differences were observed between the *Δmst3* strain and wild type (data not shown).

### Roles of *mst2* in vegetative growth and development

**Figure 2:**
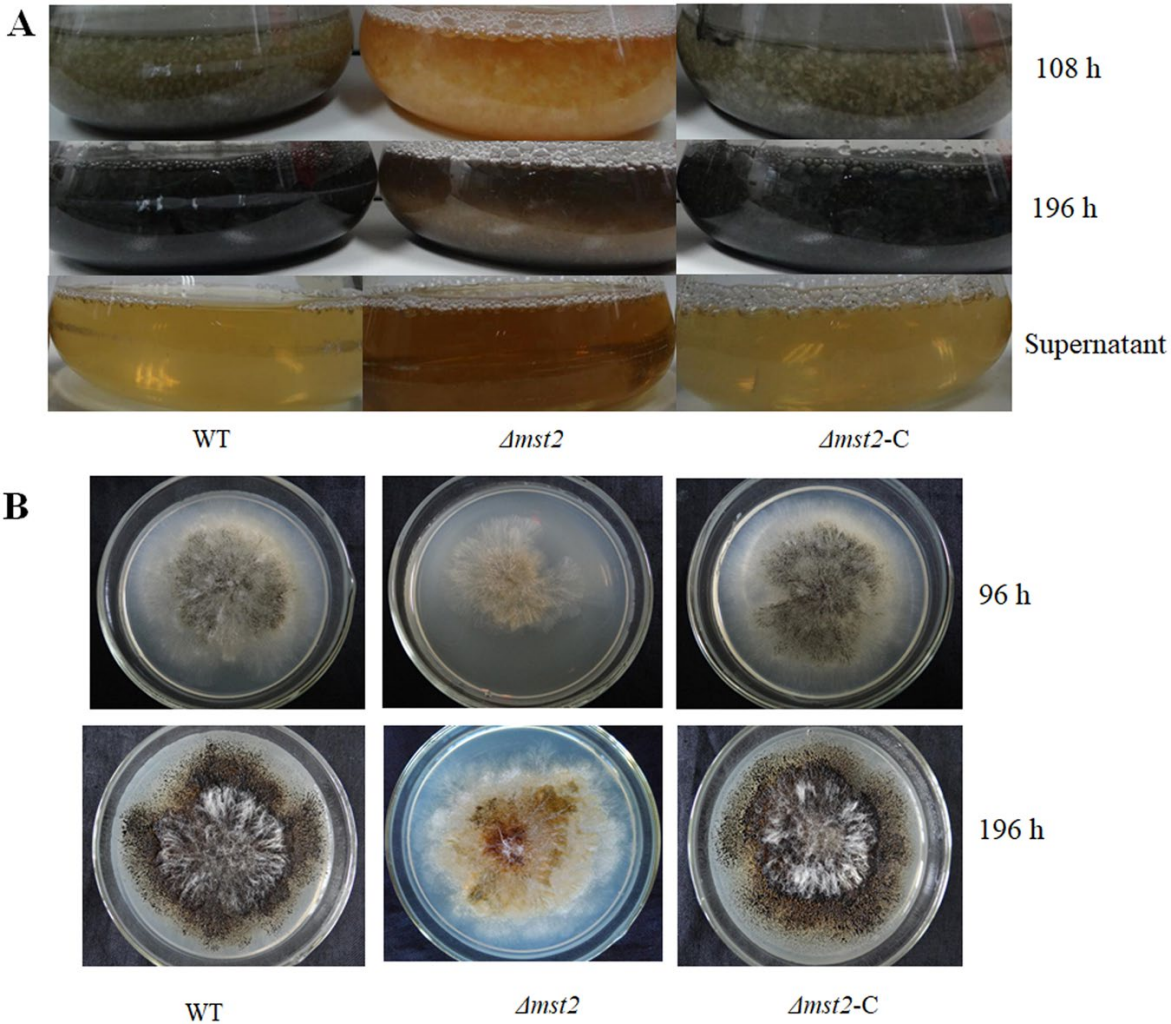
Phenotypic characterization of WT (wild type), *Δmst2* and *Δmst2*-C. (**A**) The deficiency of *mst2* delayed pigmentation, which was associated with conidiation. When cultured in liquid medium, conidia were observed in both the wild type and complemented strain after 108 h of cultivation, as indicated by the dark colour of the culture. Little conidia were seen in *Δmst2* mutant even at 108 h. (**B**) Delayed pigmentation/conidiation was also confirmed on the plates. The colonies of *Δmst2* on plates at 96 h showed less pigment (conidia by microscopy) than the wild-type and complement strains. After 196 h of incubation, conidia and pigment were generated by the *Δmst2* mutant, but at much lower levels than in the wild type. Complementation of *mst2* could restore this defective phenotype (right panels).

**Figure 3:**
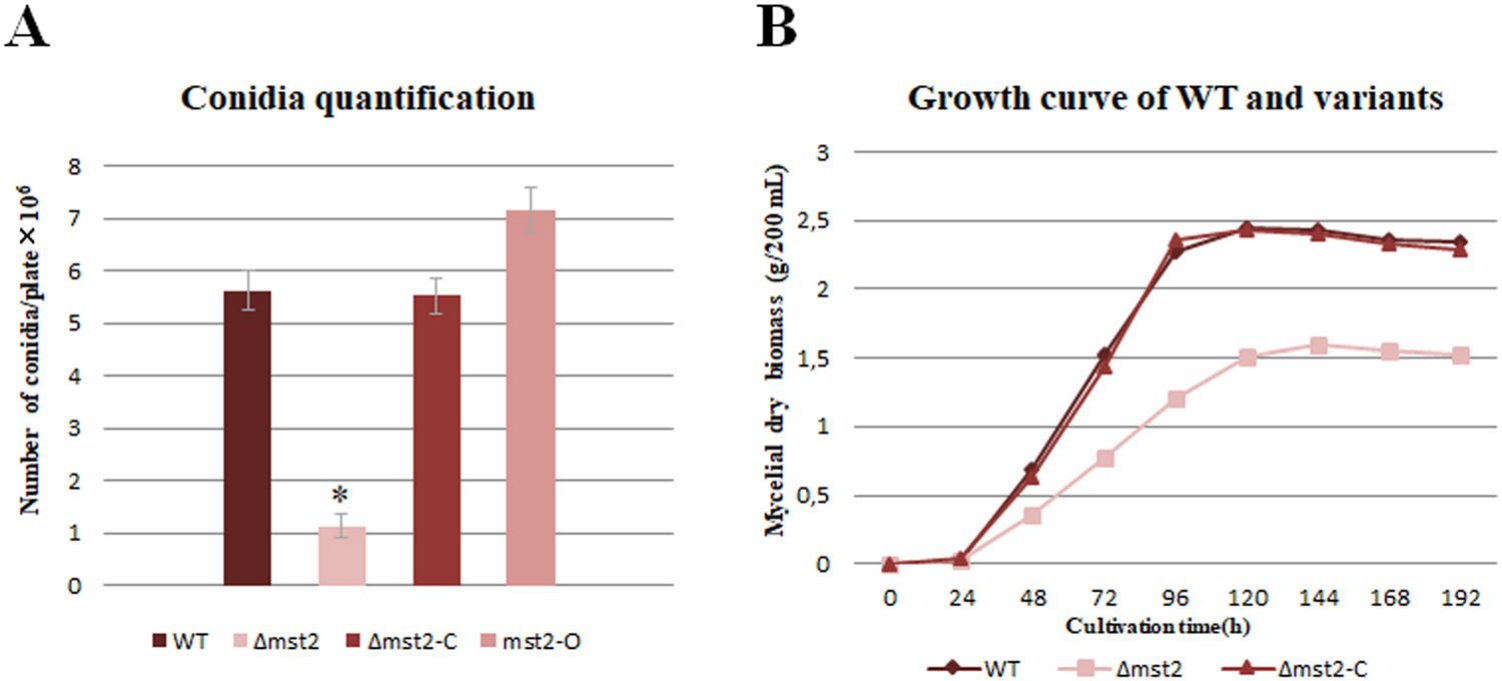
(**A**) Conidia production in WT, *Δmst2*, *Δmst2*-C and *mst2*-O was 5.61 ± 0.34 × 106 (p < 0.01), 1.13 ± 0.22 × 106 (p < 0.01), 5.52 ± 0.35 × 106 (p < 0.01) and 7.15 ± 0.45 × 106 (p < 0.01) conidia per plate, respectively. Triplicate PLA plates were incubated at 28 °C for 196 h. Error bars represent the standard deviation of triplicates. The differences were considered statistically significant at p < 0.05 (*) using the Student’s t-test. (**B**) Growth curves for WT, *Δmst2*, and *Δmst2*-C based on dry biomass. The samples were collected at the indicated time points from liquid culture. The biomass of the *Δmst2* mutant decreased by 35% relative to that of the wild type. The values are the mean of each triplicate.

To functionally characterize Mst2p in *P. microspora* NK17, we targeted the single copy of genomic *mst2* by replacing the coding region with the marker *ura3*. Candidate mutants were screened and confirmed by PCR and Southern blot (Fig. S1). First, we found that conidiation was remarkably delayed in the disrupted strain *Δmst2* (Fig. 2A). The wild-type strain usually starts to produce conidia at approximately 96 h in liquid culture, whereas little conidia was observed in *Δmst2* culture even after 120 h of cultivation, which was further supported by the same phenotype observed on solid growth medium (Fig. 2B). Complementation of *mst2* in the mutant strain by transforming back the wild-type copy of *mst2* was able to restore this conidiation defect. Apart from delayed conidiation, conidial production also dramatically decreased in *Δmst2*. Quantification analysis showed that the number of conidia produced by the wild type was 5.61 ± 0.34 × 10^6^ per plate (p < 0.01), whereas *Δmst2* produced much less at only 1.13 ± 0.22 × 10^6^ per plate (p < 0.01). The complemented strain recovered to 5.52 ± 0.35 × 10^6^ per plate (p < 0.01) (Fig. 3A). Meanwhile, vegetative growth was also affected according to a biomass reduction of approximately 35% compared to the wild type (Fig. 3B). These results suggested critical roles of Mst2p in regulating both the conidiation programme and vegetative growth in *P. microspora* NK17.

**Figure 4:**
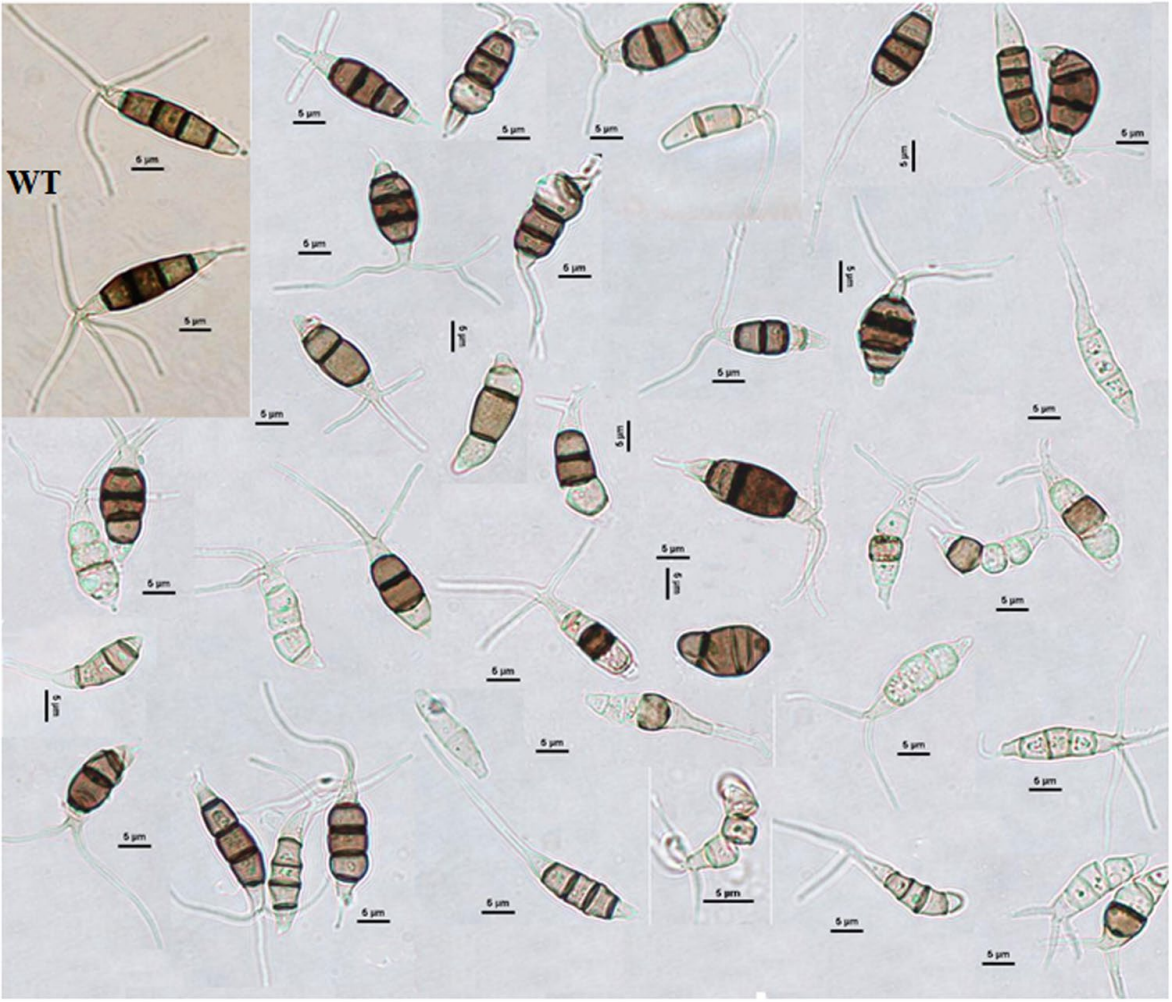
Conidia morphological variation in the mutant strain *Δmst2*. The wild-type conidia (WT, top left corner) of *P. microspora* NK17 are fusiform with four septa that form five cells. Both the terminal cells are hyaline, whereas the three median cells are brown to dark with melanin pigment. The conidia produced by *Δmst2* exhibit aberrant morphology. Scale bar, 5 µm. All conidia were collected from solid plates at 8 days.

We further examined whether the morphology of conidia was affected by the disruption of Mst2p. Wild-type conidia are fusiform and composed of four septa and five cells (Fig. 4). The basal and terminal cells are hyaline, and the median cells are pigmented with a dark colour (Fig. 4). A number of conidia produced by the *Δmst2* mutant displayed aberrant morphology (approximately 13% in liquid culture and 50% on plates by counting). As shown in Fig. 4, aberrant conidia had different cell shapes and sizes, such as only three or four cells and shortened, elongated or branched appendages. Some conidia showed different patterns of pigmentation or no pigmented cells. The irregular morphologies observed in *Δmst2* mutant conidia convincingly suggested that Mst2p is required for conidia formation, which corresponds to the temporal expression of *mst2* in the wild type.

**Figure 5:**
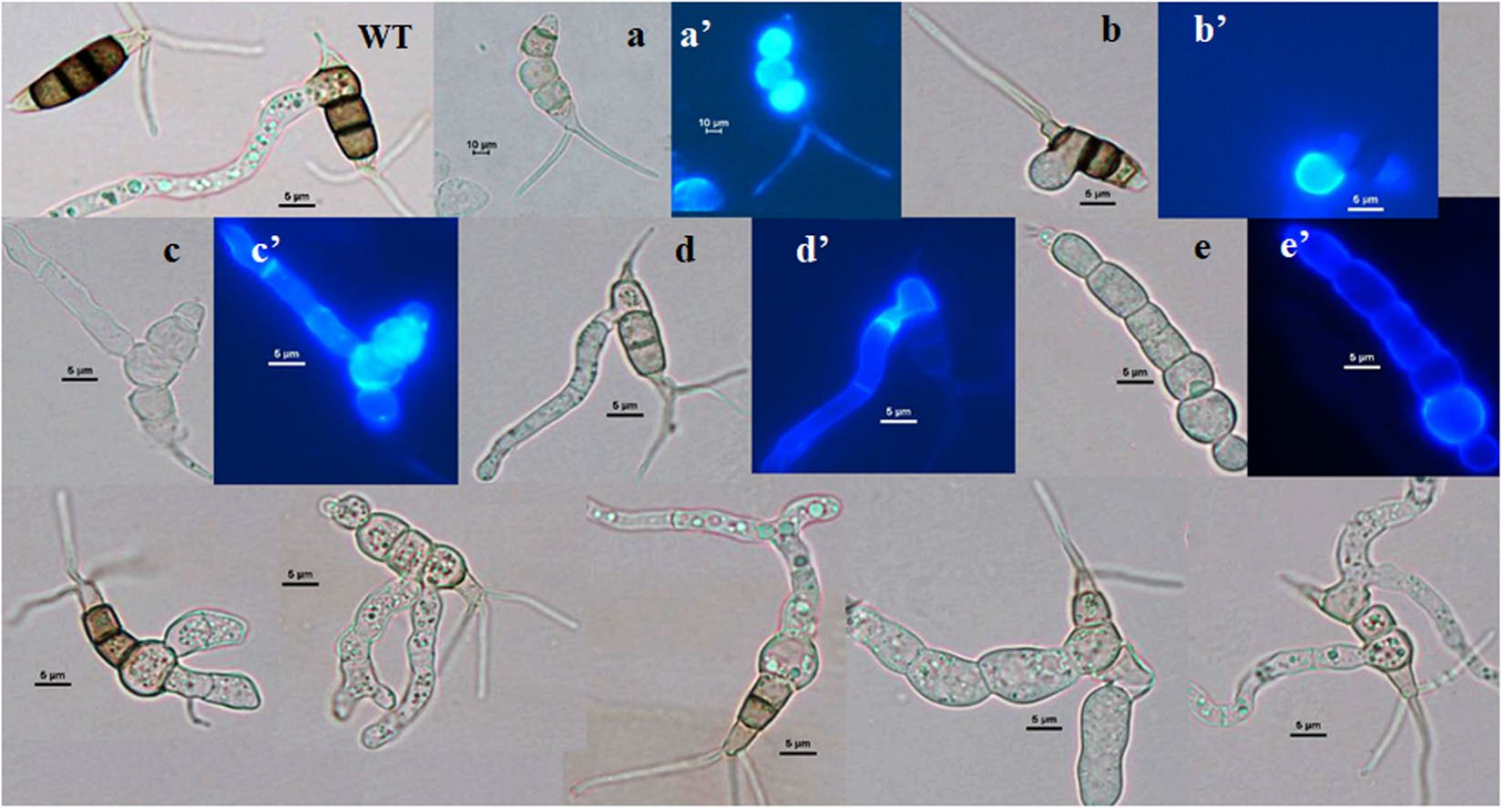
Germination of conidia from the wild type and *Δmst2* under light microscope and Calcofluor white staining fluorescence microscopy. The germination of wild-type conidia (top left corner) starts usually from the lower median cell of conidia and generates the hyphal tube. In contrast, the manner of germination was dramatically altered in the conidia from *Δmst2*. The conidia without melanin in the median cells (**a**) and the germinating cells (**b**,**c**,**d** and **e**) could take up the dye Calcofluor white (a’, b’, c’, d’ and e’), whereas the three pigmented median cells in the wild type did not^26^. Conidia were harvested from 8-day-old cultures and suspended in phosphate buffer to stain with Calcofluor white for 5 min in dark. Scale bar, 5 µm.

Following the observation of conidia development, we examined the effect of *mst2* on conidial germination process. A previous study by our laboratory suggested that *P. microspora* conidial germination normally started from the basal pigmented cell; then, a single germ tube was developed from one side, followed by the other side, which burgeoned to hypha^27^. Interestingly, an irregular germination pattern occurred in the *Δmst2* mutant (Fig. 5). Mutant conidia could germinate not only from the basal pigmented cell but also from any other cells as well, for instance, from the top median cell (Fig. 5b), the middle cell (Fig. 5c), or the basal cell (Fig. 5e), and they even germinated from two cells simultaneously (Fig. 5). This finding suggests that the disruption of *mst2* causes a loss of control of conidia germination.

### Mst2p regulates the transcription of conidiation-specific genes and the melanin gene *pks1*

**Figure 6:**
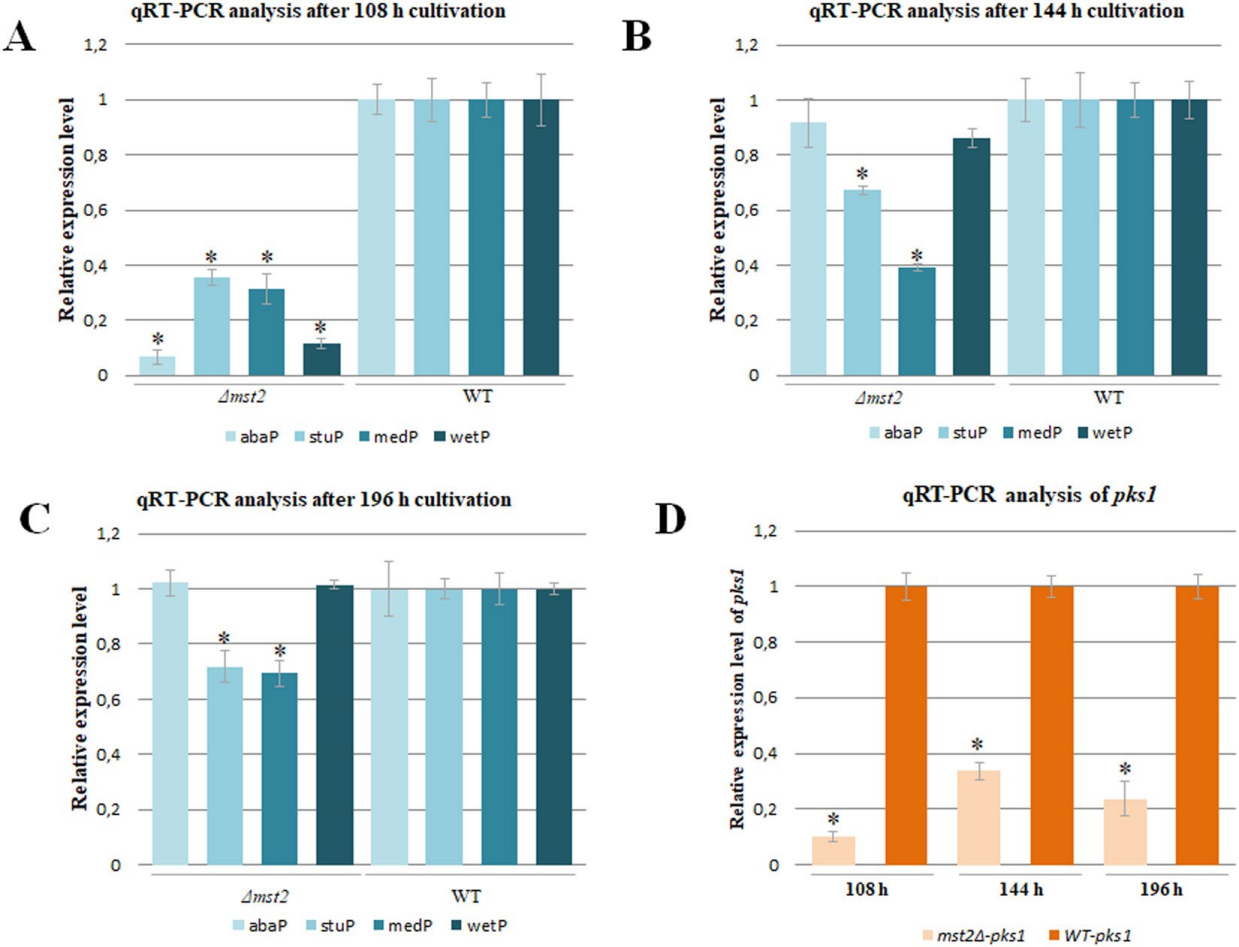
Quantitative real-time PCR analysis of the expression of the conidiation-associated genes *abaP*, *stuP*, *medP* and *wetP* (**A**,**B** and **C**), and *pks1* (**D**). Error bars represent the standard deviation of triplicates in the amplification. The differences were considered statistically significant at p < 0.05 (*) using the Student’s t-test.

To further elucidate how *mst2* affects conidiation, the expression of four putative conidiation-associated genes in *P. microspora*, *abaP, stuP, medP* and *wetP*, which are homologous to *abaA, stuA, medA* and *wetA* in *A. nidulans*^28^, were analysed through quantitative real-time PCR (qRT-PCR). In *A. nidulans*, *abaA* controls phialide differentiation^29^, *wetA* is required for the synthesis of crucial cells in conidia development^30^, and *stuA* and *medA* have been shown to be necessary for the precise spatial pattern seen in the wall components of the multicellular conidiophore^31,32^. qRT-PCR data showed that at 108 h, when conidiation started in the wild type, but not in the mutant, the transcriptional levels of *abaP, stuP, medP* and *wetP* in *Δmst2* were significantly lower than those in the wild type (Fig. 6A), suggesting that these four genes were regulated by Mst2p in *P*. *microspora*. With longer cultivation (up to 144 h), gene expression increased to some extent in the mutant, but it was still lower than wild type expression (Fig. 6B). At the end of this experiment (196 h), the expression levels of *abaP* and *wetP* in the mutant reached the same levels that were observed in the wild type, whereas the expression of *stuP* and *medP* remained at a lower transcription level (Fig. 6C). The qRT-PCR data suggest that Mst2p may act through regulating the expression of genes that are involved in conidia development, morphogenesis and germination.

We previously observed that *pks1*, a polyketide synthase gene for melanin biosynthesis in this fungus, is critical for its conidia formation^27^. The mutant *Δmst2* generated many melanin-deficient conidia (Figs 4 and 5a), which was similar to the phenotype of melanin-deficient mutant resulting from the deletion of *pks1*^27^. Therefore, it was intriguing to examine whether *pks1* was also under the control of Mst2p. We carried out qRT-PCR to determine the level of *pks1* mRNA. As shown in Fig. 6D, the expression of *pks1* in the *Δmst2* mutant was significantly reduced compared to its expression in the wild type. We conclude that Mst2p affects the melanin synthesis via controlling the transcription of *pks1* in *P. microspora* NK17.

### Cell wall integrity was compromised in the *Δmst2* mutant

**Figure 7:**
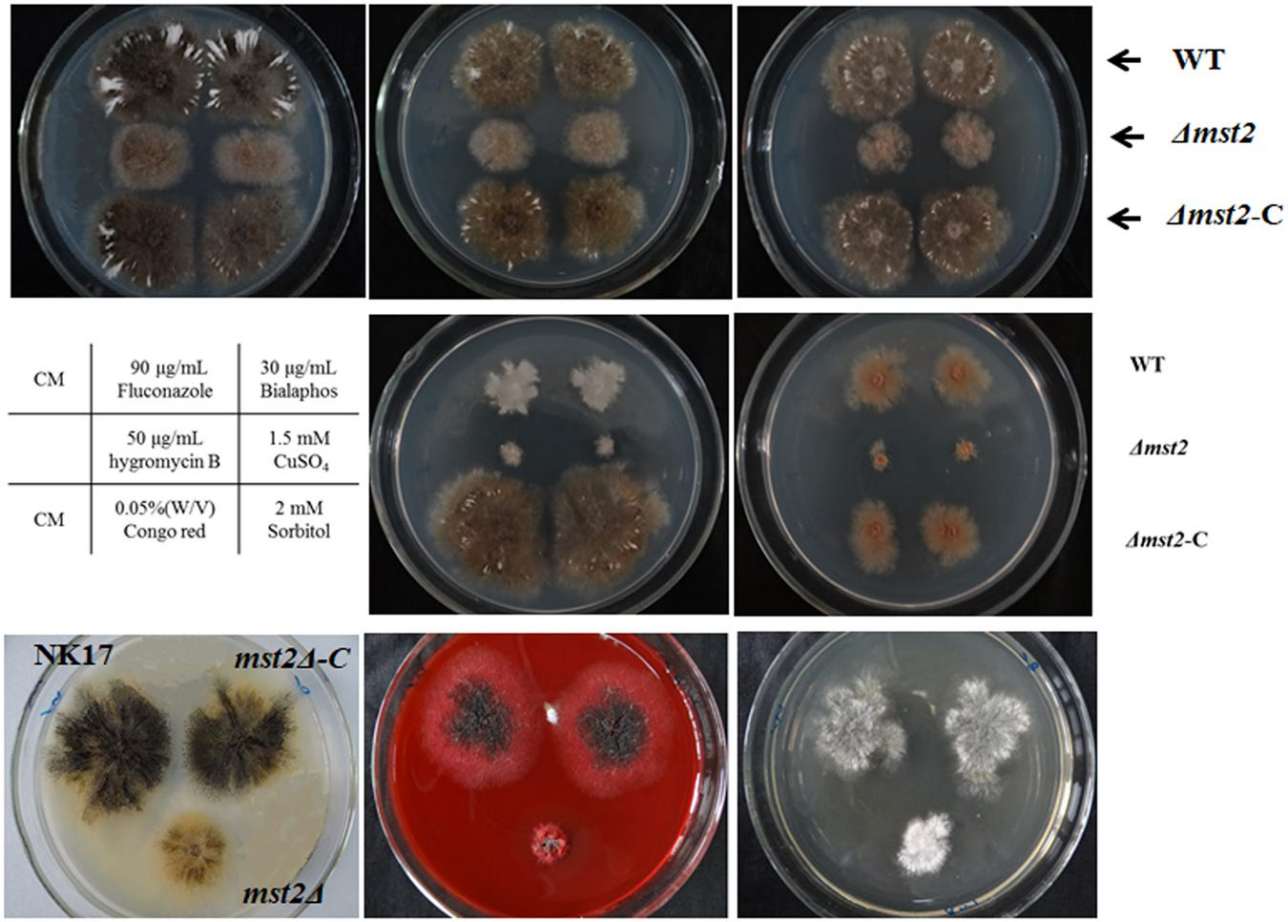
Susceptibility assay on the chemical agents. CM media was used in the assay supplemented with fluconazole, bialaphos sodium, hygromycin B, CuSO4 and external oxidative stress, Congo red and sorbitol (for concentrations and conditions, see the text). CM without chemicals served as the control. The layout of strains on Congo red and sorbitol plates is shown on the control plate (down left) or indicated on the right (WT, *Δmst2* and *Δmst2*-C).

Several HAT proteins in fungi have been proven to play a role in tolerance to stress conditions, *e.g*., oxidative agents, inhibitors, antibiotics and heavy metals. To test whether MYST Mst2p also influences the response of *P. microspora* to stress, we performed assays mimicking stress conditions by separately supplying the media with one of the following chemicals: fluconazole, bialaphos sodium, hygromycin B, CuSO_4_, SDS, sorbitol or Congo red. Interestingly, the fungi (WT, *Δmst2* and *Δmst2*-C) showed resistance to the antifungal drug fluconazole and the herbicide bialaphos, which had little effect on growth (Fig. 7). On the other hand, all strains could hardly grow on plates supplemented with hygromycin B, CuSO_4_ or SDS (not shown) (Fig. 7). A significant finding of these tests is that *Δmst2* showed high sensitivity to Congo red and sorbitol, which is normally used to check cell wall integrity in fungi^33,34^ (Bottom panels, Fig. 7). While our knowledge about the cell wall structure of *P. microspora* NK17 is still limited, this result suggests that cell wall integrity is impaired by the inactivation of *mst2*.

### Crucial roles of *mst2* in the biosynthesis of secondary metabolites

Fungal secondary metabolism and sporulation are often temporally and functionally related^35–37^. Both processes have been demonstrated to share some common regulatory elements. To further explore the putative roles of *P. microspora* Mst2p in secondary metabolite production, we conducted a profiling analysis for secondary metabolites (SMs) in different strains by High Performance Liquid Chromatography (HPLC). SM production usually commences at the late phase of culturing. Consistently, in *P. microspora*, SMs in the wild type were hardly detectable at time points before 96 h (Fig. S3), and they usually reached a maximum at approximately 196 h.

**Figure 8:**
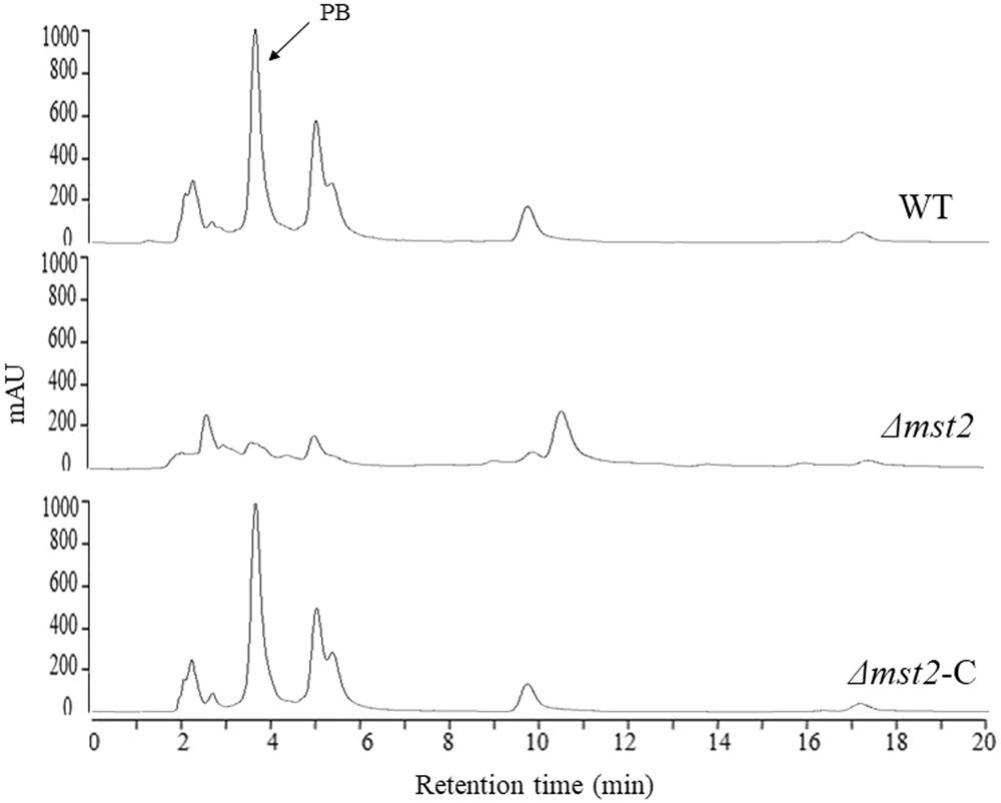
HPLC profiling for the detection of secondary metabolites was prepared from liquid cultures of the WT, *Δmst2* and *Δmst2*-C strains. Compared to WT, *Δmst2* showed a dramatic decrease in SM production. Pestalotiollide B (PB) is highlighted. The SM production in *Δmst2*-C was recovered by complementation of the wild-type *mst2* allele.

HPLC profiling of the cultures at 196 h revealed that the deletion of *mst2* resulted in a drastic reduction of SM production (Fig. 8). For instance, pestalotiollide B (PB), a structurally characterized product in this fungus^19^, was barely detectable in *Δmst2*, whereas PB production was recovered in the complemented strain *Δmst2*-C, clearly demonstrating a crucial role of *mst2* in the biosynthesis of secondary metabolites in *P. microspora*.

### Overexpression of *mst2* promotes conidiation but inhibits secondary metabolite biosynthesis

**Figure 9:**
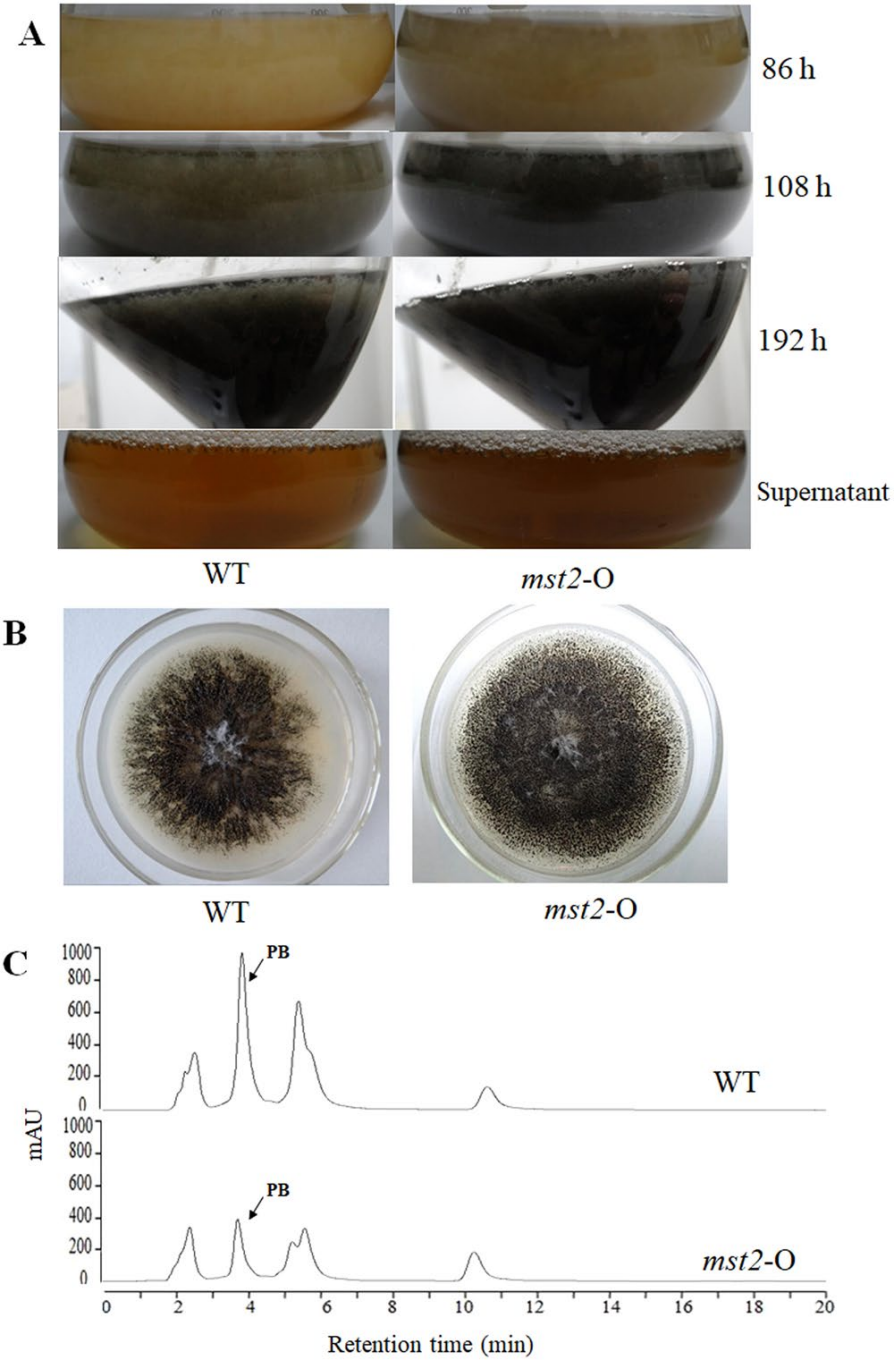
The colony phenotype and HPLC profiling for secondary metabolites in the overexpression strain *mst2*-O. (**A**) The overexpression of *mst2* promoted the conidiation process, initiating conidiation at least 10 h earlier than when it occurred in the wild type and increased conidia production as shown by the darker colour of the liquid culture. In addition, *mst2*-O appeared to produce more secreted pigment in the supernatant (bottom photos). (**B**) The morphology of *mst2*-O on PLA. (**C**) Overexpression of *mst2* depressed secondary metabolism. The biosynthesis of PB decreased by ~60%. HPLC profiling analysis was repeated three times.

Considering the positive role of *mst2* in conidiation and secondary metabolism and a previous study noting that the overexpression of another type of HAT-coding gene *esaA* can increase SM production in *A. nidulans*^14^, we overexpressed *mst2* to further examine its action in *P. microspora*. The overexpression strain *mst2*-O was generated by inserting a wild-type *mst2* allele into an *ura3*-disturbed site of NK17- *Δura3* (Fig. S2). As seen in Fig. 9A and B, the overexpression of *mst2* led to earlier conidiation, approximately 10 h earlier than when it occurs in the wild type in liquid shaking culture. In addition, conidia production increased in the *mst2*-O strain both in liquid culture and on plates by ~27.45% (Figs 3A and 9B). The promotion of conidiation caused by *mst2* overexpression supported the earlier demonstration of *mst2* in the conidiation process.

Unexpectedly, overexpression of *mst2* resulted in a lower yield of SMs in the mutant strain (Fig. 9C). This result suggests that normal expression of Mst2p is necessary for the proper regulation of secondary metabolism.

## Discussion

*P. microspora* has received considerable attention in the past decade for its ability to produce a variety of secondary metabolites, including the anticancer drug Taxol (paclitaxel)^38^. Based on preliminary data from the genome sequence, *P. microspora* NK17 has great potential to produce SMs, *e.g*., there are 48 genes that putatively encode polyketide synthetases (PKSs)^39^ and non-ribosomal peptide synthetases (NRPSs)^40–42^. To explore the regulation of secondary metabolism, we reported the characterization of three genes in *P. microspora* NK17 encoding MYST histone acetyltransferases by starting with the identification of *mst2* from a mutant library. Through a loss-of-function approach, we found that one of the genes, *mst1*, is likely to be essential for survival of the fungus, whereas the third gene, *mst3*, appears to be dispensable. Loss of *mst2* led to plural phenotypic alternations in pigmentation, conidia production, morphology and germination, drug resistance and especially the biosynthesis of secondary metabolites.

Our experimental results suggest that the main function of *mst2* is involved in the development of conidia in *P. microspora*. Disruption of *mst2* resulted in not only significant delay of conidiation and a sharp reduction in conidia number but also abnormal morphology of the conidia as well (Figs 3 and 4). The percentage of aberrant conidia produced by the mutant strain was estimated to be 13% in liquid culture or 50% on plates. In *A. nidulans*, the central regulatory pathway of conidiation encompasses three transcription factors, *BrlA*, *AbaA* and *WetA*^38^. AbaA and WetA proteins have been shown to be responsible for conidia formation and maturation. Two other developmental regulatory genes, *stuA* and *medA*, are necessary for the precise spatial pattern of multicellular conidiophores^28^. In *P. microspora*, the homologues of the four condition-specific genes (*brlA* is missing), named accordingly, *abaP*, *wetP*, *stuP* and *medP*, were down regulated in the *mst2* mutant strain, implying that *mst2* acted through the regulation of these conidiation-specific genes.

One of the interesting findings in this study is that *mst2* affected conidial germination (Fig. 5). In *P. microspora*, conidial germination is likely under the strict control of an unidentified mechanism. Germination normally begins with swelling and hyphal tube formation from the lower median cell, indicating a coordinating mechanism of action^27^. However, in the mutant strain, germination could start from any one of the conidial cells or even simultaneously from two cells, implying the loss of coordination. As we showed, the temporal expression of *mst2* coinciding with the conidiation process in the wild type (Fig. 1C) also supports this speculation. Additionally, the overexpression of *mst2* promoted conidiation, causing this process to occur at least 10 h earlier (Fig. 9A), and in the meantime, it increased conidia production (Figs 3A and 9B). This finding paves the way for studying the regulation of fungal conidia germination.

In *P. microspora*, the polyketide synthase gene, *pks1*, is responsible for melanin biosynthesis and the formation of multiple-cellular conidia^27^. Disruption of *pks1* causes a deficiency of conidial pigmentation (melanin) and the formation of five-cellular conidia but is dispensable for vegetative growth. In the *Δmst2* mutant, the expression of *pks1* was significantly down-regulated throughout the time points examined (Fig. 6D). Concomitantly, many conidia exhibited entire or partial melanin deficiency in the *Δmst2* strain (Figs 4 and 5a). Unlike the *pks1* deletion mutant, the body cells of the conidia stayed together^27^, suggesting there is basal expression of *pks1* in *Δmst2*. Moreover, in the wild type, the three pigmented-median cells of the conidia are usually unable to be stained by Calcofluor white. Rather, the conidia of *Δmst2* could be stained by the dye, suggesting that the permeability of the conidial cell wall was impaired. This behaviour is in line with the phenotype of *Δpks1*^27^. Another finding related to cell wall integrity is that in the stress sensitivity assay, *Δmst2* showed high sensitivity to Congo red and sorbitol (Fig. 7), also suggesting cell wall damage in the mutant^26^. According to this result, we speculate a critical role of *mst2* in the biogenesis of the cell wall.

Importantly, we found that mst2 was required for the production of secondary metabolites in *P. microspora*. HPLC profiling disclosed a significant reduction of most if not all secondary metabolites in the mutant strain *Δmst2* (Fig. 8). For example, PB production decreased 7-fold in *Δmst2* (Fig. 8B). This remarkable change in SM production suggests an indispensable role of *mst2* in secondary metabolism in the fungus. However, contrary to what we expected, overexpression of *mst2* did not increase SM production; rather, it resulted in an overall reduction in secondary metabolism (Fig. 9C). The findings may help with identification of the pathways and genes involved in the processes in this fungus by approaches such as RNA-seq or proteomics analysis.

Our work still suggests that MYST family member Mst2p in *P. microspora* may function as a general effector responding to upstream signals associated with conidiation, vegetative growth and secondary mechanism. Given that the classic G-protein-mediated growth pathway in *A. nidulans* and *P. microspora* regulates growth, asexual sporulation and natural product biosynthesis^26,35,43^, it is intriguing to speculate that the G-protein-mediated pathway may exhibit cross-talk with MYST Mst2p in filamentous fungi concerning growth, conidiation and secondary metabolism.

## Materials and Methods

### Strains and culture conditions

*P. microspora* and the bacterial strains used in this study are listed in Table S1. *P. microspora* NK17 was originally isolated as a Taxol-producer and stored in our laboratory (China Patent ZL 2008 10152500.1). Fungi were grown on Potato Lactose Agar (PLA) or in PLB without agar usually at 28 °C. Luria Bertani (LB) medium was used for bacterial culture at 37 °C. Induction medium (IM) and yeast nitrogen base (YNB) with 2% lactose were used for *Agrobacterium tumefaciens*-mediated transformation (ATMT). The media and culture conditions for ATMT are described in detail in the following sections.

### Vector construction

Construction of the vectors for gene deletion, complementation and overexpression were all based on a protocol called OSCAR described by Paz *et al*.^44^. The vector for targeted deletion, pOSCAR-*mst2*, was generated in 5 µl of BP clonase reaction, which contained 60 ng of pA-Ura3-OSCAR^45^, 60 ng of pOSCAR (Invitrogen, CA, USA), 20 ng of 5′ and 3′ flanking fragments of *mst2* and 1 µl of BP clonase II enzyme (Invitrogen, CA, USA). The *mst2* 5′ and 3′ flank fragments were obtained by PCR amplification of the NK17 genome with primer pairs Mst2-up(F)/Mst2-up(R) and Mst2-down(F)/Mst2-down(R), respectively (Fig. S1A).

For complementation, the plasmid pOSCAR-*mst2-*C carrying the wild-type copy of *mst2* was constructed from pA-Hyg-OSCAR^45^, pOSCAR, *mst2* allele and *ura3* as a selection marker. The *mst2* allele and *ura3* genes were obtained by PCR amplification of the NK17 genome with primer pairs Mst2-up(F)/Mst2-down(CR) and Ura3(F)/Ura3(R), respectively (Fig. S1A). The overexpression plasmid pOSCAR-*mst2-*O was created from parts of the pA-Ura3-OSCAR, pOSCAR + *ura3*-up, *mst2* allele (acting as 5′ flank) and *ura3*-down (acting as 3′ flank). Briefly, pOSCAR + *ura3*-up was derived from pOSCAR through enzyme digestion and ligation with the *ura3*-up fragment amplified with primer pair Ura3-up(F)/Ura3-up(R). The *ura3*-down fragment was cloned with primer pair Ura3-down(F)/Ura3-down(R). This overexpression plasmid allowed the *mst2* allele to be inserted at the *ura3* locus in the NK17-*Δura3* strain (Fig. S2). All primers in this study are listed in Table S2. All resulting plasmids were verified by restriction enzyme digestion and sequencing.

### Transformation procedure

The plasmids pOSCAR-*mst2*, pOSCAR-*mst2-*C or pOSCAR-*mst2-*O were individually transformed into the *A. tumefaciens* LBA4404 strain using the heat-shock method^27^. The fungal transformation described previously by Yu *et al*.^27^ was applied with minor modifications. For gene knockout and overexpression, *A. tumefaciens* LBA4404 harbouring pOSCAR-*mst2* or pOSCAR-*mst2*-O were mixed with conidia harvested from the recipient fungal strain NK17-*Δura3*. Then, the mixture was co-cultured on a nitrocellulose membrane over an IM plate (induction medium) with uracil (50 mg/L) for counterselection and acetosyringone (40 mg/L) for induction. After 48 h of incubation, the membrane was transferred onto a YNB plate containing cefotaxime (100 μg/mL) and was incubated for another 48 h. For gene complementation, the *Δmst2* strain served as the recipient. The IM plate was supplemented with only acetosyringone. PLA supplemented with cefotaxime and hygromycin B (100 μg/mL) was used for the selection plate. Individual transformants were purified by single spore isolation.

### Southern blot analysis

Transformants with site-specific disruption and complementation of the *mst2* gene were confirmed further by Southern blot analysis after an initial screening by PCR. Isolation of the *P. microspora* genome DNA was described previously by Hao *et al*.^20^. Southern blot was carried out by following the protocol provided by the supplier of DIG High Prime DNA Labeling and Detection Starter Kit II (Roche China, Shanghai, China). Double digestion of the DNA samples by the enzymes *Xho*I/*Bam*HI (Takara, Dalian, China) was conducted and separated by 0.8% agarose gel. A probe that covered *mst2*-up and the conjoint part of *mst2* ORF was amplified using the primer pair Mst2-up(F)/Mst2-orf(R), which is shown in Fig. S1.

### RNA isolation and reverse transcriptase PCR (RT-PCR) analysis

Total RNAs was extracted by TRIzol Reagent (Invitrogen, CA) from *P. microspora* mycelia grown in 200 mL of PLB at 28 °C for 2, 3, 4, 6, or 8 days with shaking at 180 rpm. The concentration of RNA samples was quantified by Biowave DNA (Biochrom, UK), and its quality was checked by agarose gel. First-strand cDNA was made with equal concentrations of total RNA using an M-MLV RTase cDNA Synthesis Kit (Takara, Dalian, China), following the manufacturer’s instructions, and then this cDNA was used in PCR amplifications with the primer pair Mst2-orf(F)/Mst2-orf(R). All transcripts were normalized with the transcript quantities of the actin gene (*act1* in NK17^27^) as a reference. PCR amplifications were performed in triplicate.

### Quantitative real-time PCR (qRT-PCR) to evaluate gene expression

qRT-PCR was performed on an ABI StepOne Real-Time PCR System (Applied Biosystems, USA) following the protocol of the Fast Start Universal SYBR Green Master (Roche) according to the manufacturer’s instructions. The fluorescent signal obtained for each gene was normalized with the mRNA of the actin gene *act1*. Triplicate amplification was performed for each sample and analysed by the Ct (2^−ΔΔCt^) method.

### Phenotype characterization

Conidia of *P. microspora* were harvested from liquid culture in PLB at 28 °C for 8–10 days with shaking, and then their concentration was determined by haemocytometry. Biomass was harvested by vacuum filtration from liquid cultures collected at a series of time points and then freeze-dried to determine the dry weight for the growth curves. Conidia germination assay was performed as described by Yu *et al*.^27^. Calcofluor white (CFW) staining (Sigma, St. Louis, USA) was adopted in the observation of conidial morphology. Photos were taken under UV illumination by fluorescence microscopy by a Nikon Eclipse 80i fluorescence microscope (Nikon Inc, Japan).

Stress sensitivity was tested on complete medium (CM) supplemented separately with the following chemicals: fluconazole (30 and 60 μg/mL, final concentration), bialaphos sodium (5, 15 and 30 μg/mL), hygromycin B (30, 50 and 80 μg/mL), CuSO_4_ (0.5 and 1.5 mM), SDS (0.07% (w/v)), sorbitol (0.05% (w/v)) and Congo red (2 M).

### Secondary metabolite profiling by HPLC

Secondary metabolite production was determined by general profiling with HPLC. Strains were grown in 200 PLB for 8 days with shaking at 180 rpm. Mycelium and the liquid phrase were separated by vacuum filtration with 3 M filter paper. Extraction of secondary metabolites was conducted with a previously reported procedure^19^ with slight modification. Briefly, the culture supernatant was mixed with an equal volume of dichloromethane thoroughly and then set statically overnight. The organic phase was condensed by vacuum-rotary evaporation until it was dry. The dried residue was dissolved in 1 mL of methane. After filtration with a Millipore filter (0.45 μm), 10 µl of the suspension was subject to profiling by HPLC (Agilent 1100, Agilent Technologies, Santa Clara, CA) following the programme described^46^.

## Electronic supplementary material

Supplementary file
