# Supplementary material for: A MYST Histone Acetyltransferase Modulates Conidia Development and Secondary Metabolism in Pestalotiopsis microspora, a Taxol Producer
Source: Sci Rep. 2018 May 29;8:8199. doi: 10.1038/s41598-018-25983-8 (PMC5974303; doi:10.1038/s41598-018-25983-8)
Supplement: Supplementary file 1 — Supplementary file [file 41598_2018_25983_MOESM1_ESM.pdf]

A MYST Histone Acetyltransferase Modulates Conidia Development and Secondary Metabolism in *Pestalotiopsis microspora*, a Taxol Producer

Qian Zhang[1#], Oren Akhberdi[1#], Dongsheng Wei[1], Longfei Chen [1], Heng Liu[1], Dan Wang[1], Xiaoran Hao[2], Xudong Zhu[2*]

[1] State Key Program of Microbiology and Department of Microbiology, College of Life Sciences, Nankai University, Tianjin 300071, P.R. China

[2] Beijing Key Laboratory of Genetic Engineering Drug and Biotechnology, Institution of Biochemistry and Molecular Biology, College of Life Sciences, Beijing Normal University, Beijing 100875, P.R. China

[#] These authors contributed equally to this work.

[*] Corresponding author: Xudong Zhu, Institution of Biochemistry and Molecular Biology, College of Life Sciences, Beijing Normal University, Beijing 100875, P.R. China. E-mail address: zhu11187@bnu.edu.cn

Table S1 Fungal and bacterial strains used in this study

Stain	Genotype	Reference
Pestalotiopsis microspora NK17	Wild type	Laboratory stock
Agrobacterium tumefaciens LBA4404	TiAch5	1
NK17-*Δura3*	*ura3*::none	2
Escherichia coli DB3.1	F^- *gyr*A462 *end*A1 Δ(sr1-*rec*A) *mcr*B *mrr* *hsd*S20(r_B^-, m_B^-) *sup*E44 *ara*-14 *gal*K2 *lac*Y1 *pro*A2 *rps*L20(Str^R) *xyl*-5 λ^- *leu mtl*1, *ccd*B Survival™ 2	Invitrogen, CA
Escherichia coli DH5α	F– Φ80*lac*ZΔM15 Δ(*lac*ZYA-*arg*F) U169 *rec*A1 *end*A1 *hsd*R17 (rK–, mK+) *phoA sup*E44 λ– *thi*-1 *gyr*A96 *rel*A1	Invitrogen, CA
Δmst2	*mst2*::*hygR*	In this study
Δmst2-C	*Δmst2*::*mst2*	In this study
mst2-O	NK17-*Δura3*::*mst2*	In this study

1. Hellens, R., Mullineaux, P. & Klee, H. Technical focus: a guide to Agrobacterium binary Ti vectors. *Trends Plant Sci.* **5**(10), 446-451. (2000).
2. Chen, L. F. *et al.* Orotidine 5′-phosphate decarboxylase-based reusable in situ genetic editing system: Development and application in taxol-producing *Pestalotiopsis microspora*. *Eng. Life Sci.* **15**,542–49 (2015).

Table S2 Primers used in this study

Primer	Primer sequence 5’→3’
Mst2-up(F)	GGGGACAGCTTTCTTGTACAAAGTGGAAAGACCTTGCAGTCTCACGAA
Mst2-up(R)	GGGGACTGCTTTTTTGTACAAACTTGTTGTTGGCTCACTTGGTACTA
Mst2-down(F)	GGGGACAACTTTGTATAGAAAAGTTGTTTGTGACTTCAACGCCTCC
Mst2-down(R)	GGGGACAACTTTGTATAATAAAGTTGTATGTGGGCGAATCAAGAC
Mst2-down(CR)	GGGGACTGCTTTTTTGTACAAACTTGTATGTGGGCGAATCAAGAC
Mst2-orf(F)	CCTCAGGATGGCGAAGTA
Mst2-orf(R)	CGACAGTATCGCCCAGTT
Mst2-uofu(F)	CCTTATCCGACTGTCCTCTC
Mst2-dofd(R)	TTCGATTCAGAATGGGGCAC
Ura3-orf(F)	GTCAAGACATCTGTTACCGTGG
Ura3-orf(R)	AGTCCTTGTGCTCCCGTG
Ura3-up(F)	GGGGTACCCCGTCGTGTGTAGGTGGGTAAA
Ura3-up(R)	AGGGCCCTATGGGGCTCTGTCGTTCTAT
Ura3-down(F)	GGGGACAACTTTGTATAGAAAAGTTGTTAAGGCGGCATGTGATTGT
Ura3-down(R)	GGGGACAACTTTGTATAATAAAGTTGTGGTGTGTCTTTGGTCGTGT
Ura3(F)	GGGGACAACTTTGTATAGAAAAGTTGTTGAACGACAGAGCCCCATC
Ura3(R)	GGGGACAACTTTGTATAATAAAGTTGTTTCACTGGCAAGTAGGTA
Hyg(F)	GCCCTTCCTCCCTTTATT
Hyg(R)	TGTTGGCGACCTCGTATT
Actin1(F)	CATTGAGCACGGTGTTGT
Actin1(R)	TCTTCTCACGGTTGGACTT
Pks1(F)	CCGCAAGGCTACATCGGTAC
Pks1(R)	TGAAAGGCGAGAAGAGTCCC
AbaP(F)	TCGGAAACCTTTGTAATGC
AbaP(R)	TACCGTTGAGGAGAAGATGA
MedP(F)	TGACTGAGGGCTGGACTA
MedP(R)	CACTGACTGCCTTGAAACT
StuP(F)	TCAGCCATACGATAACTCACG
StuP(R)	CTTCACATCTTGCTCACCC
WetP(F)	CCTCCTTCTTTGACTTTCC
WetP(R)	TCTCCTCTTTGGGCGACT

Underlined text in primer sequences represent the attB recombination sequence added to the 5’ end of each primer[33], except for Ura3-up(F)-*Kpn*I and Ura3-up(R)-*Apa*I.

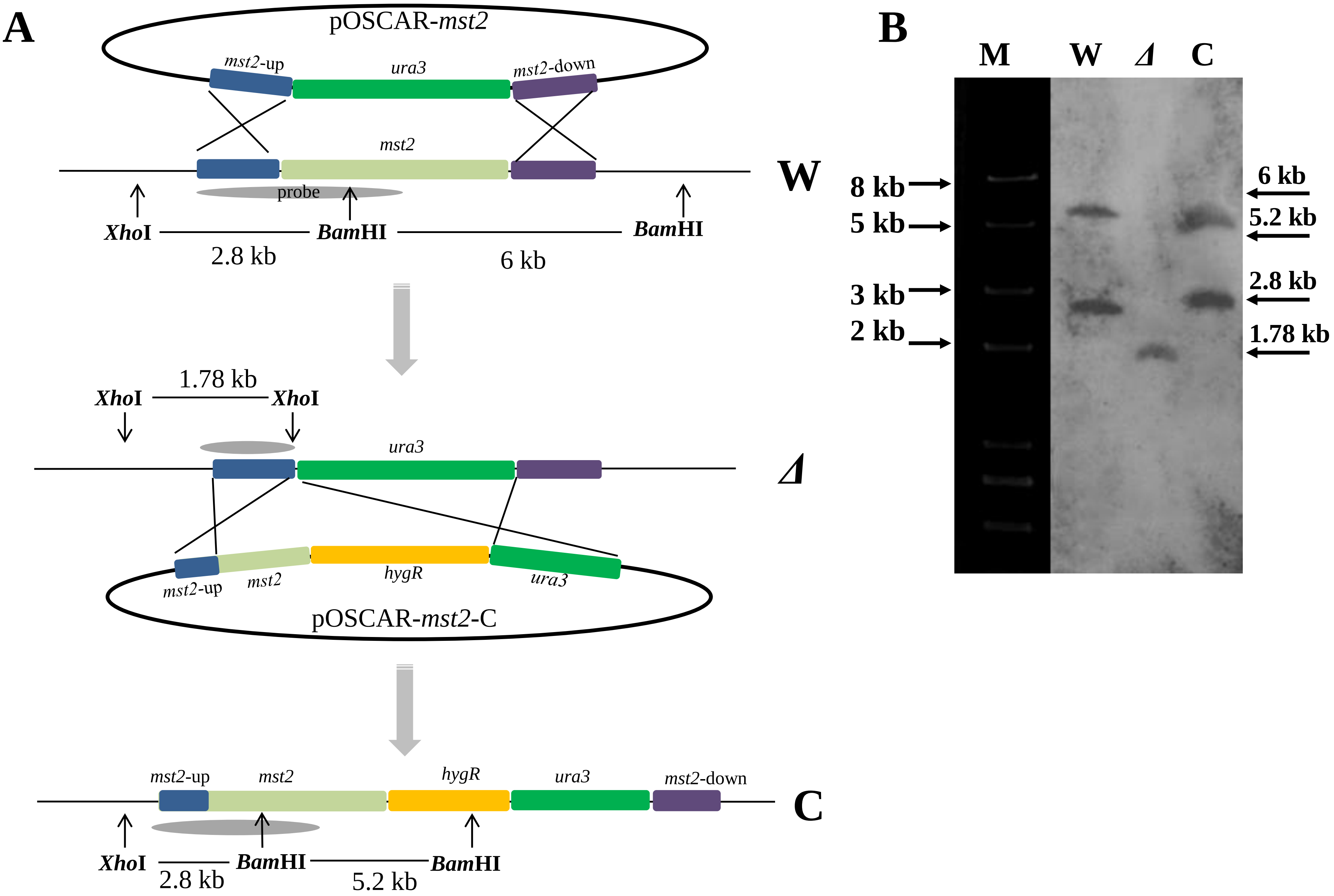
A
pOSCAR-mst2
mst2-up
ura3
mst2-down
mst2
W
probe
XhoI
BamHI
BamHI
2.8 kb
6 kb
1.78 kb
XhoI
XhoI
ura3
Δ
mst2-up
mst2
hygR
ura3
pOSCAR-mst2-C
mst2-up
mst2
hygR
ura3
mst2-down
C
XhoI
BamHI
BamHI
2.8 kb
5.2 kb
B
M
W
Δ
C
8 kb
5 kb
3 kb
2 kb
6 kb
5.2 kb
2.8 kb
1.78 kb

Figure S1.

Fig. S1 Construction of *mst2* deletion and complementation strains. (A) Diagrams showing the strategy for generating *mst2* deletion and complementation strains by homologous recombination. The probes used for Southern blot hybridization, the location of the recognition sites for enzymes *Xho*I and *Bam*HI used to digest fungal genomic DNA are indicated. Predicted sizes of the hybridization bands for each strain are shown. (B) Southern blot hybridization to confirm the deletion and complementation of *mst2*. The genomic DNA from the wild-type (W), *Δmst2* (*Δ*) and *Δmst2*-C(C) strains were digested by *Xho*I and *Bam*HI. The DIG-labelled probe bounded to two bands, 6 kb and 2.8 kb respectively, in the wild-type and a single 1.78 kb band in *Δmst2*, indicating the replacement of *mst2* by *ura3*. A 5.2 kb and a 2.8 kb band were detected in *Δmst2*-C, suggesting the successful *in situ* complementation of *mst2* in deletion strain. M refers to Trans2K plusII (TransGen, China) molecular weight marker.

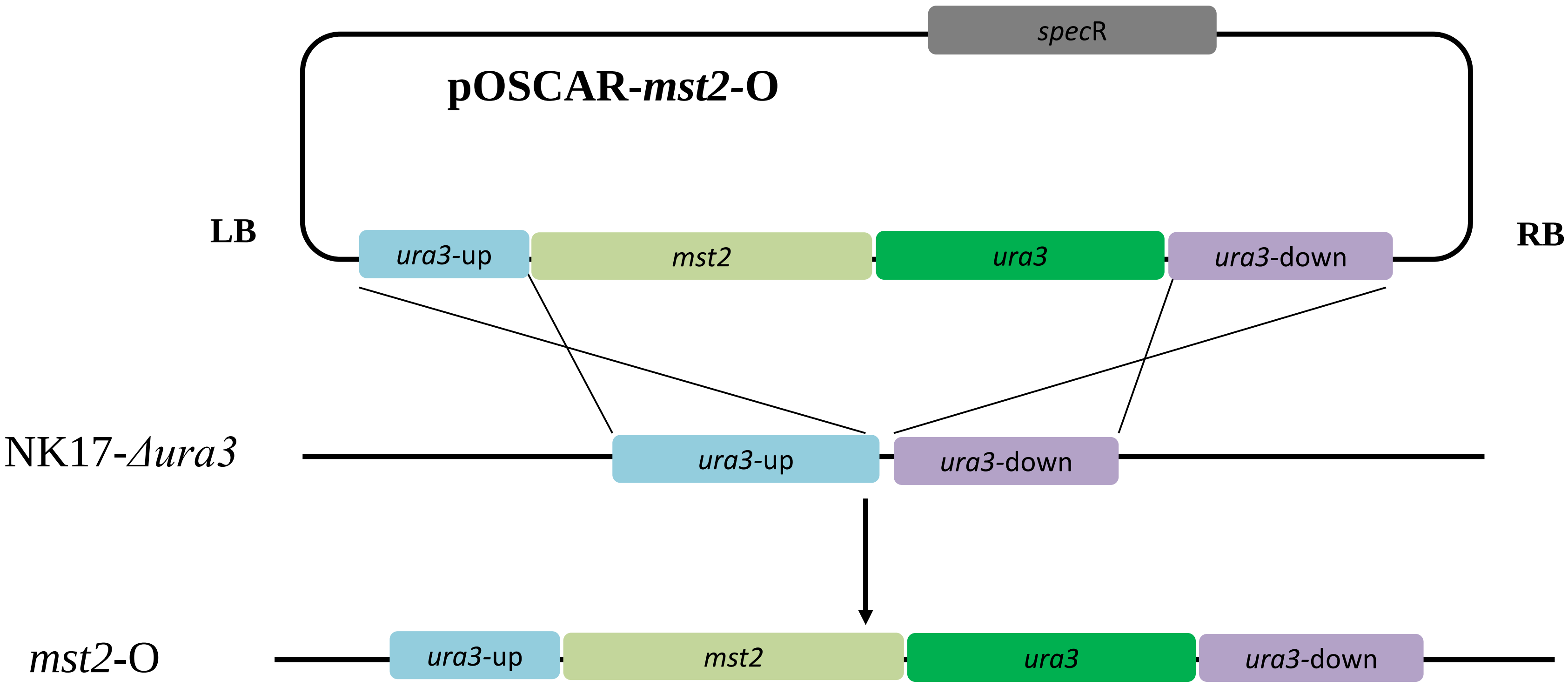

Figure S2. Diagrams showing the strategy for generating *mst2* overexpression strains by homologous recombination in NK17-*Δura3* strain.

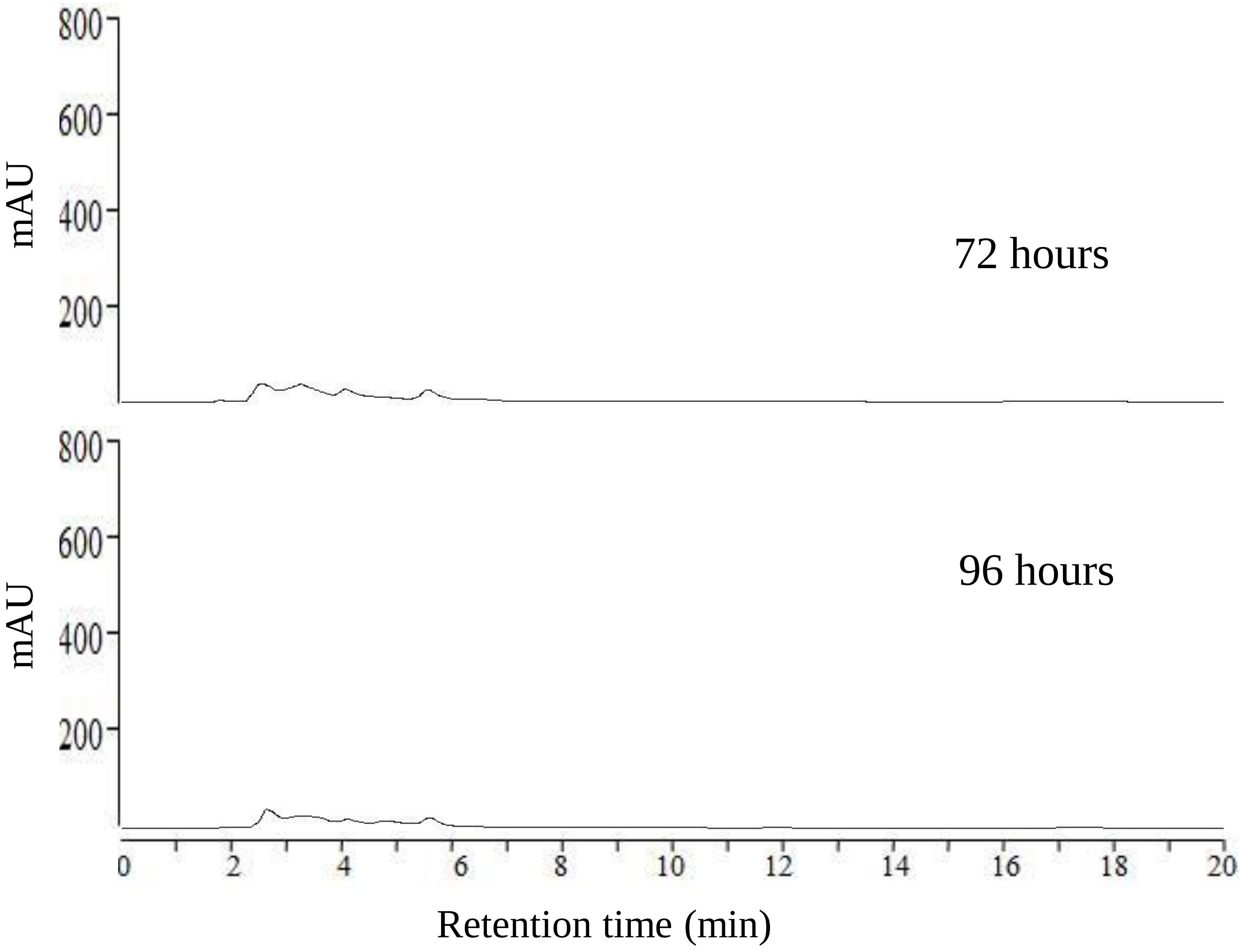

Figure S3. HPLC analysis of the SM in NK17 after cultivation for 72 hours and 96 hours. Almost nothing can be extracted from fermentation liquor, except very few pigment from PLB medium.

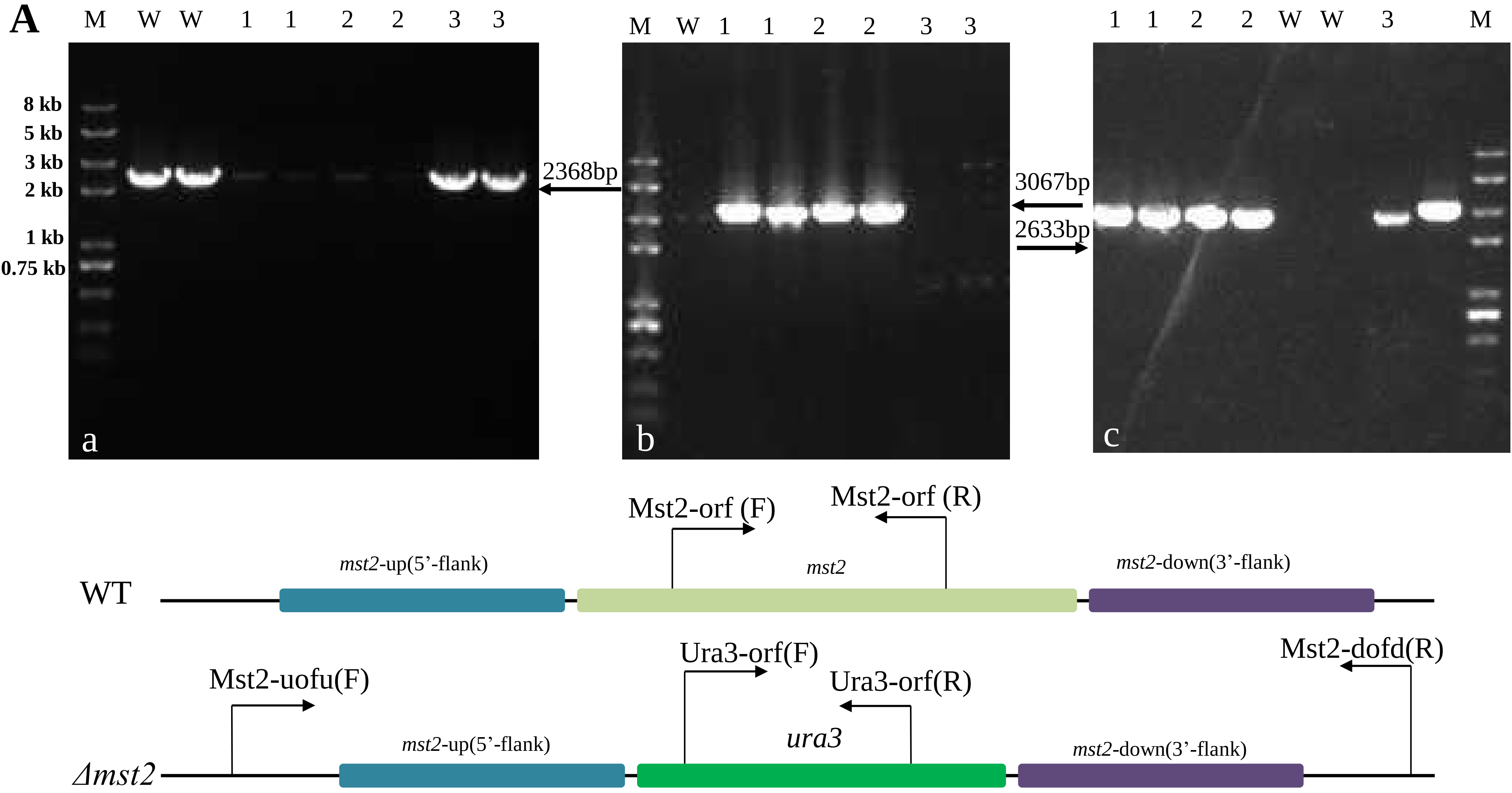

Figure S4A. Characterization of deletion strain *Δmst2* by PCR purification. (a) Confirmation of the *mst2* ORF using primer pair Mst2-orf(F)/Mst2-orf(F), a 2368 bp band can be obtained in wild-type, but not for right mutants; (b and c) Confirmation of the replacement of *mst2* by *ura3* marker. A 3067 bp and a 2633 bp bands can be amplified with Mst2-uofu(F)/Ura3-orf(R) and Ura3-orf(F)/Mst2-dofd(R), respectively, in right mutants, while no bands in wild-type. The letters: W above the lane represents wild-type strain, M represents the molecular weight marker (Trans2K plusII, TransGen, China). Numbers (1-3) above the lane refer to the analyzed *P. microspora* transformants. The sites of the primers were shown in the schematic representation of wild-type and mutant.

B

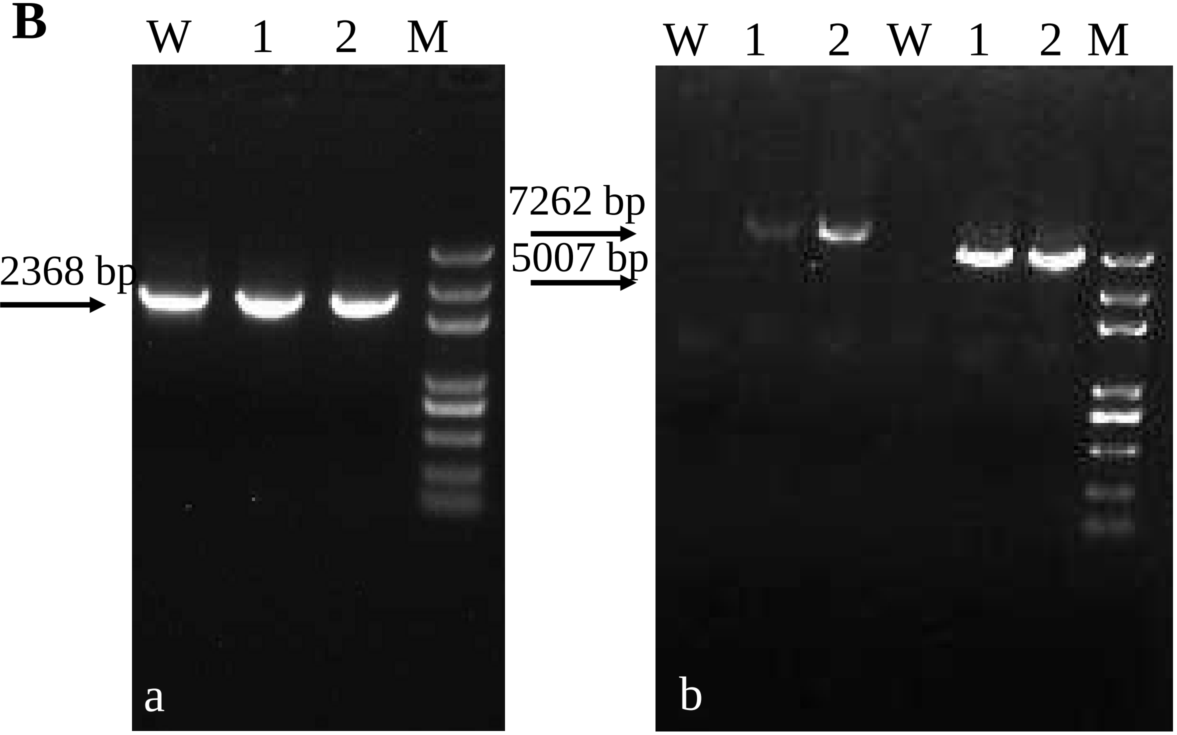

Figure S4B. Characterization of complementation strain *Δmst2*-C by PCR purification. (a) Confirmation of the *mst2* ORF using primer pair Mst2-orf(F)/Mst2-orf(F). Wild-type and right mutants can generate a 2368 bp band. (b) Further confirmation of the complementation of *mst2 in situ* using primer pairs Mst2-uofu(F)/Hyg-orf(R) and Hyg-orf(F)/Mst2-dofd(R), in which a 7262 bp and a 5007 bp bands can be generated, respectively, in right mutants, while no bands in wild-type. Numbers (1-2) above the lane refer to the analyzed transformants. The sites of the primers were shown in the schematic representation of wild-type and complementation strain *Δmst2*-C.

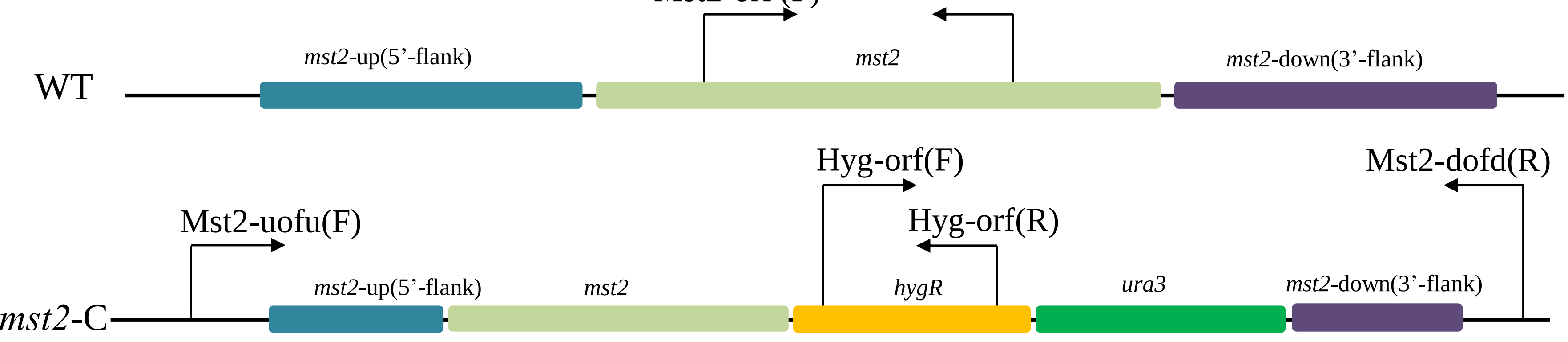

C

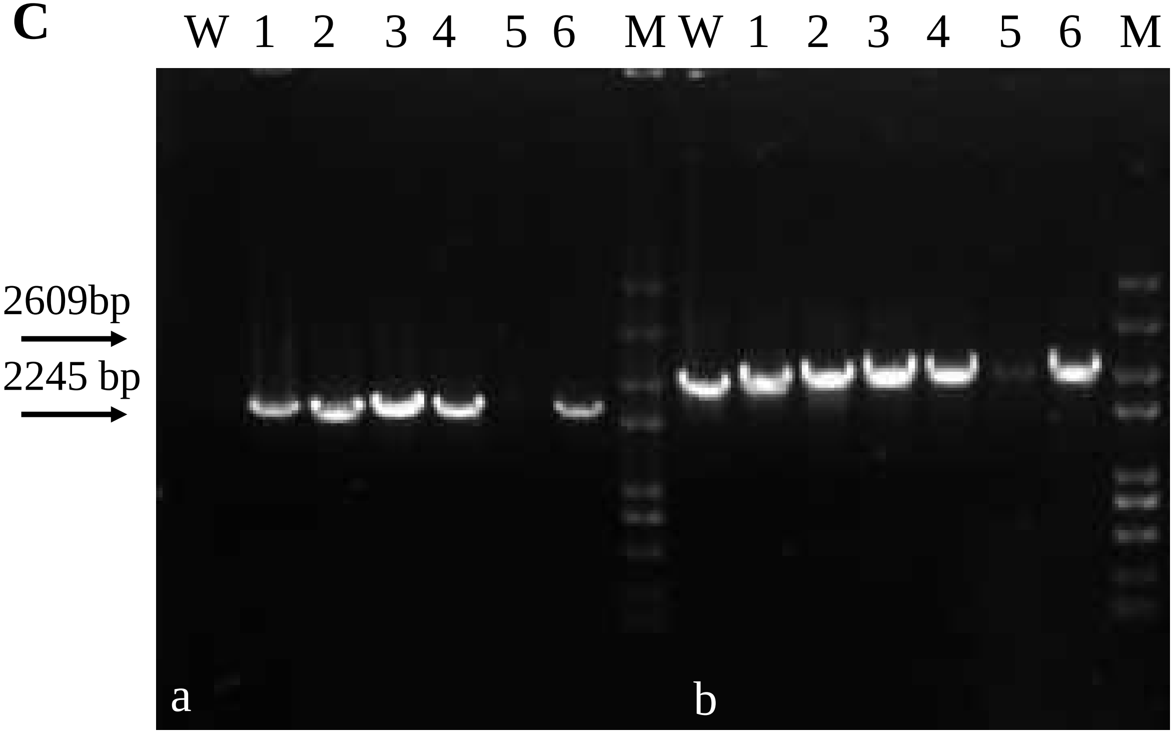

Figure S4C. Characterization of overexpression strain *mst2*-O by PCR purification. Primer pairs Ura3-uofu(F)/Mst2-orf(R) and Ura3-orf(F)/Ura3-dofd(R) were used to check *mst2* allele was rightly inserted in *ura3* site in NK17-*Δura3*. A 2245 bp band (a) can be generated with Ura3-uofu(F)/Mst2-orf(R) in right mutants, but not in wild-type, while a 2609 bp band (b) can be generated with Ura3-orf(F)/Ura3-dofd(R) both in wild-type and right mutant. Numbers (1-6) above the lane refer to the analyzed transformants. The sites of the primers were shown in the schematic representation of overexpression strain *mst2*-O.

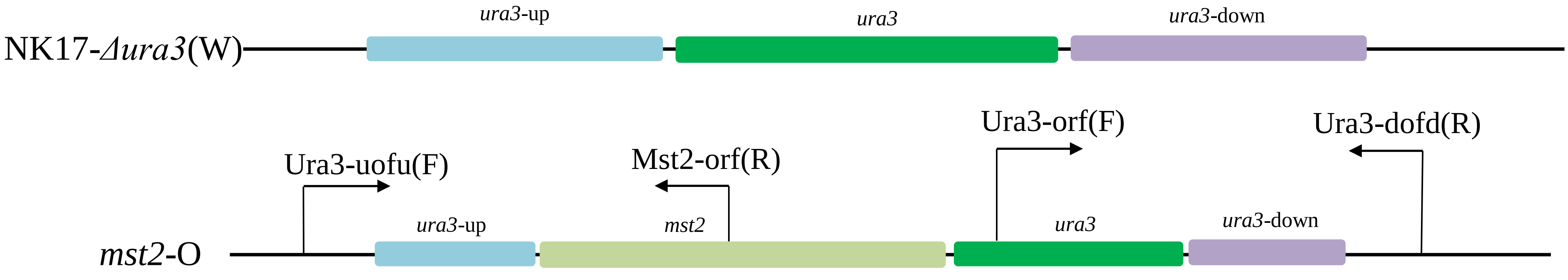

A Fluconazole

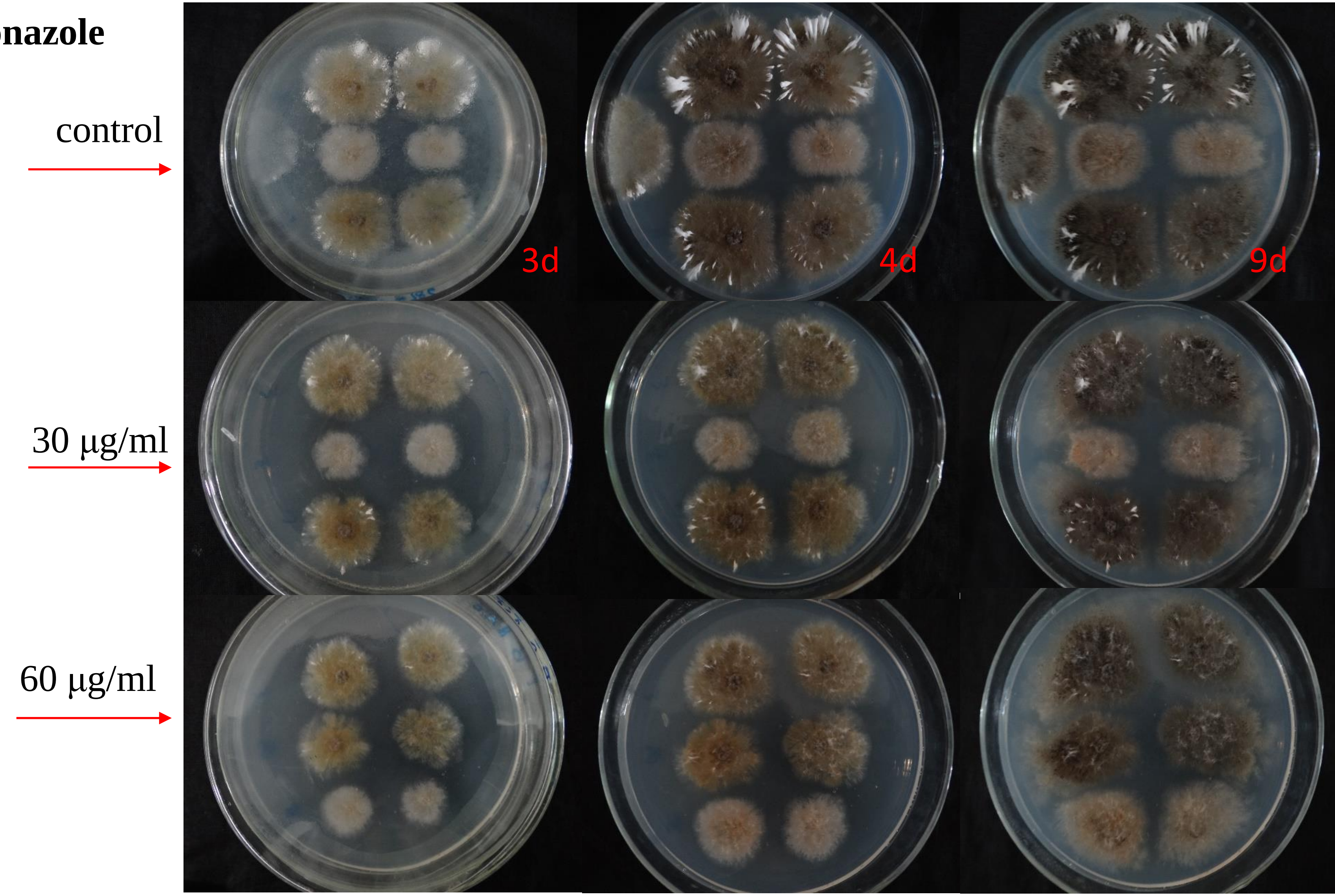

Figure S5A

B Bialaphos

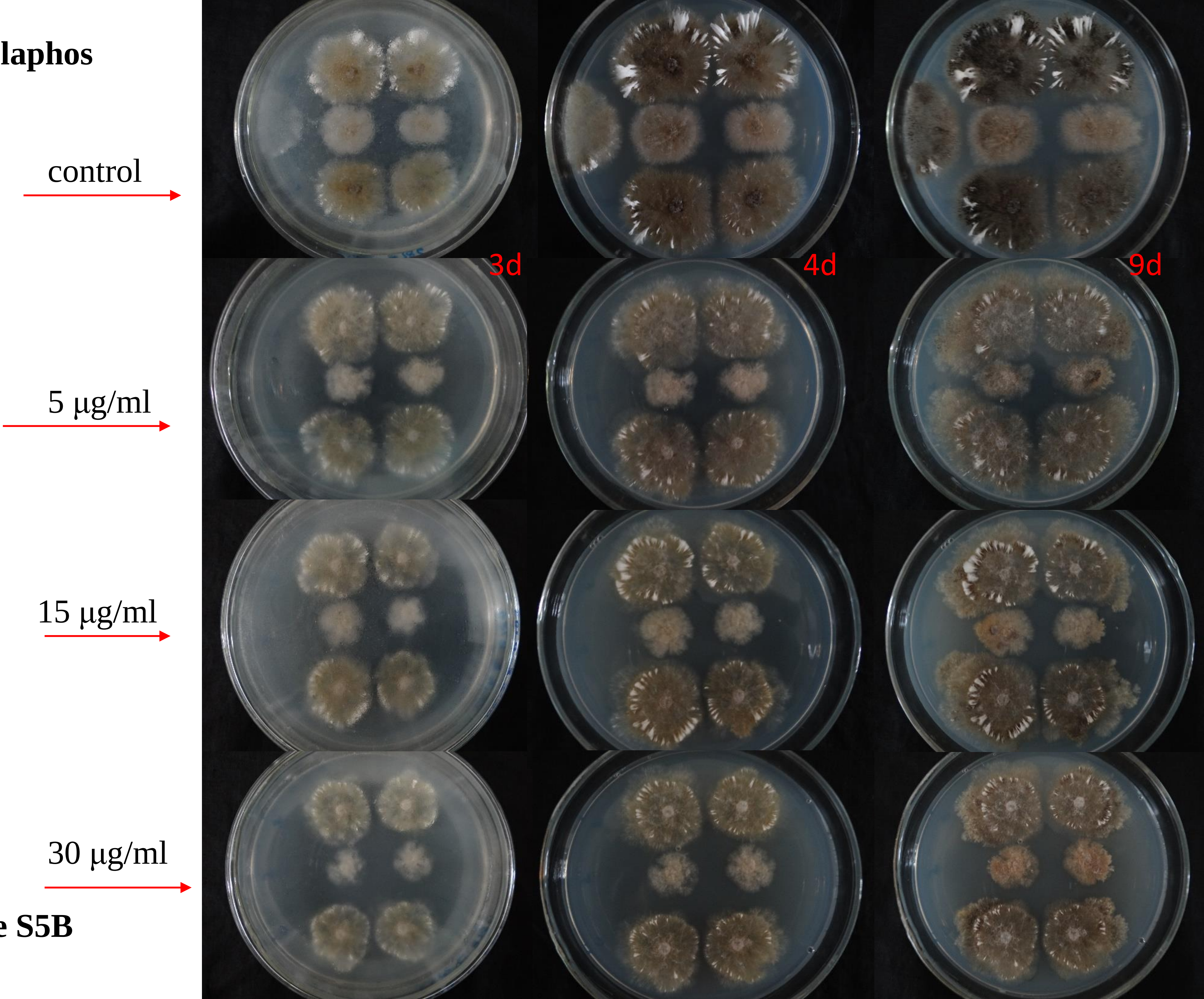

Figure S5B

C Hygromycin B

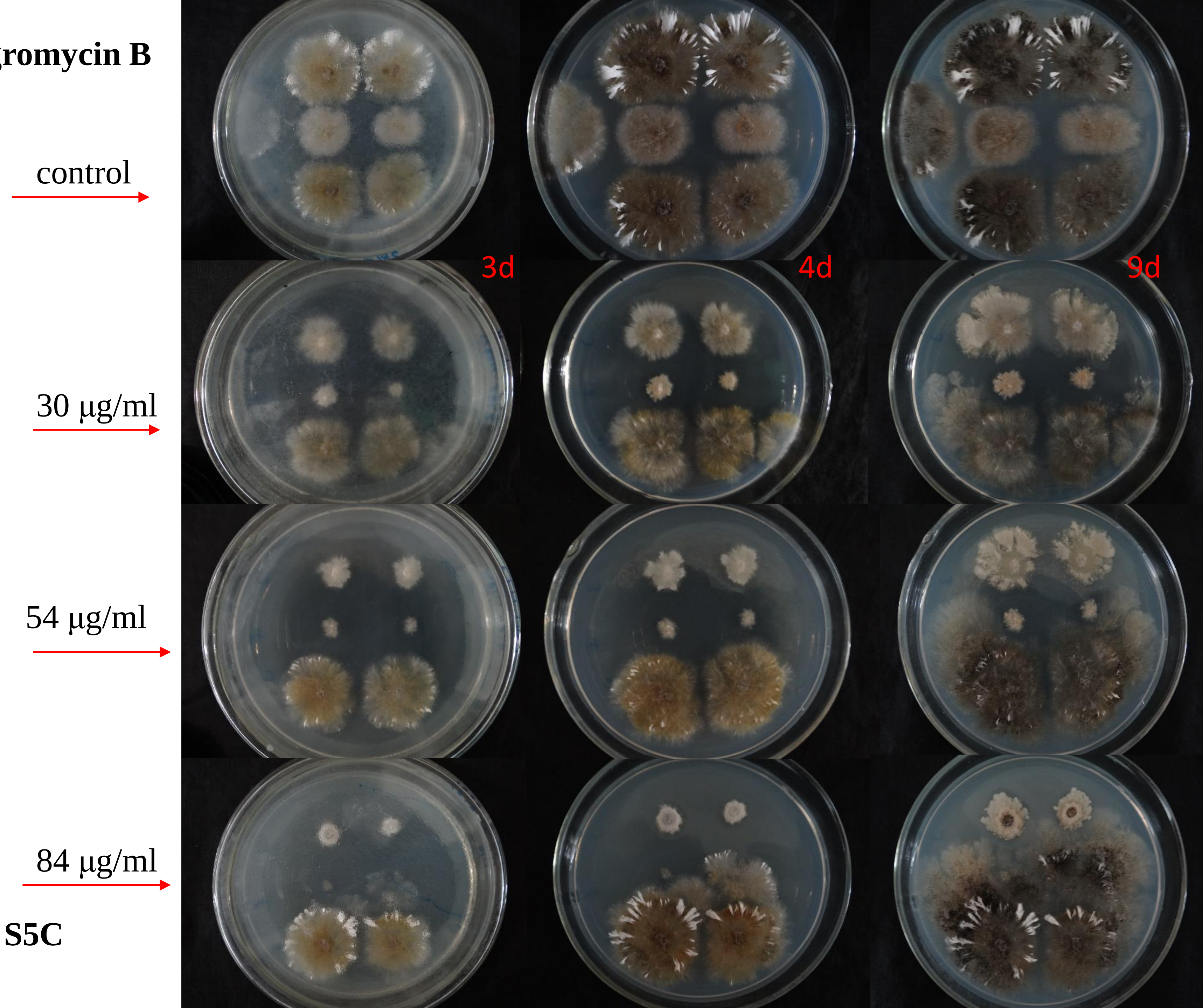

Figure S5C

D $CuSO_4$

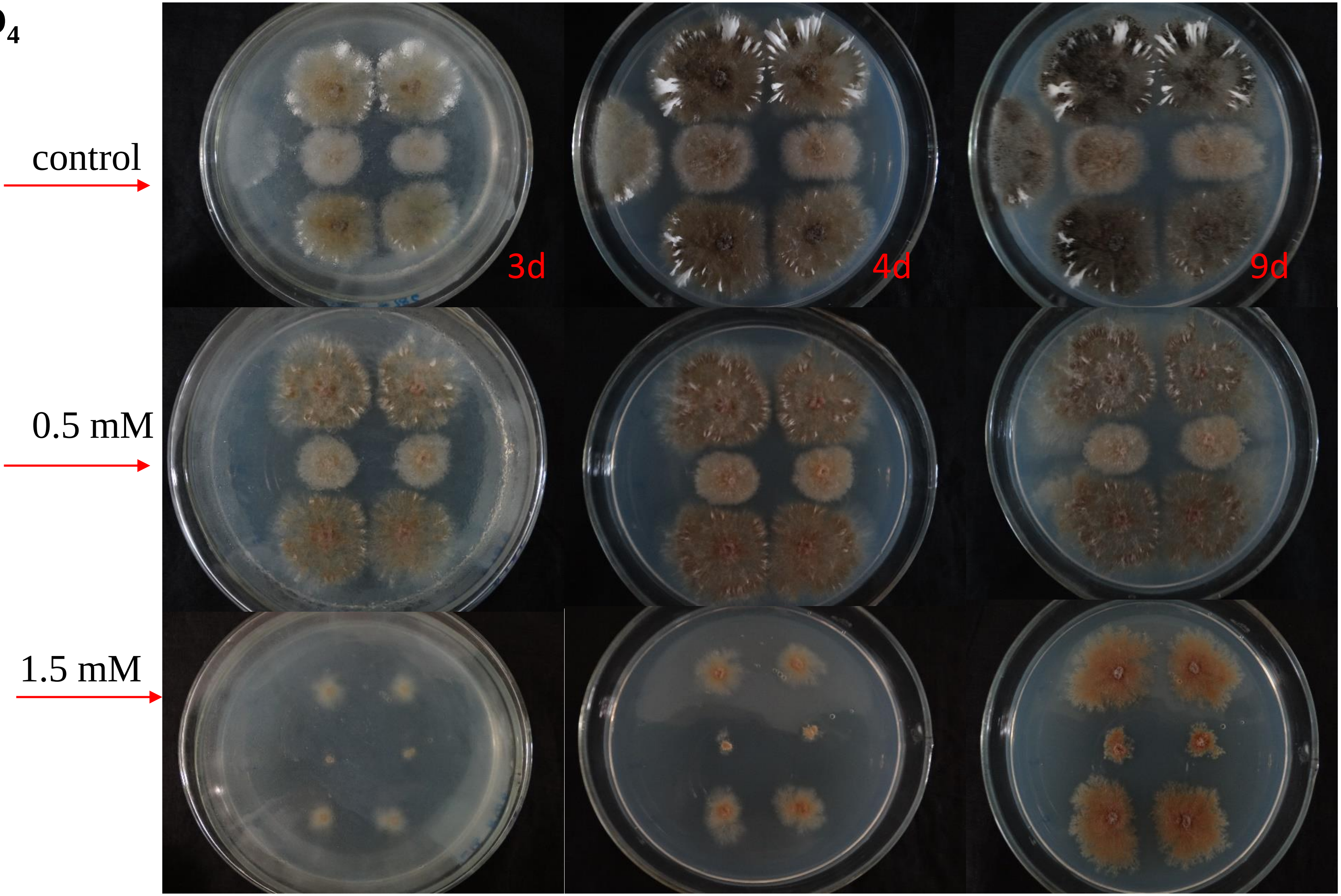

Figure S5D

Figure S5A-D. Susceptibility assay with chemical agents. The assay with fluconazole(30 and 60 μg/mL), bialaphos sodium(5, 15 and 30 μg/mL), hygromycin B (30, 50 and 80 μg/mL) and $CuSO_4$ (0.5 and 1.5 mM) were conducted on CM media. CM without any supplementary served as control(Top row on every slide). The photos were took at 3th (left lane), 4th (middle lane) and 9th (right lane) day as seen above.The layout of strains on plates was the same with it shown in Fig. 7. The *Δmst2*-C showed no inhibition to hygromycin B because of the presence of hygromycin B resistant gene in this strain as selective marker.
